# Lactylation in kidney diseases: a review of regulatory mechanisms and therapeutic prospects

**DOI:** 10.3389/fcell.2026.1836185

**Published:** 2026-07-27

**Authors:** Yuxi Feng, Jinjin Wang, Jiawei Cao, Xuan Zhang, Mengyu Liang, Yi Cai, Jiaqi Wang, Chenxi Lu, Runzhi Zhu, Qinyang Jin, Zhuanxin Chen, Qin Zhu

**Affiliations:** 1 Department of Nephrology, Hangzhou TCM Hospital Affiliated with Zhejiang Chinese Medical University, Hangzhou, China; 2 National Clinical Research Center for Child Health, The Children’s Hospital, Zhejiang University School of Medicine, Hangzhou, China; 3 HeartCenter, Department of Geriatrics, Zhejiang Provincial People’s Hospital (Affiliated People’s Hospital, Hangzhou Medical College), Hangzhou, Zhejiang, China; 4 Department of Endocrinology, Chun’an County Traditional Chinese Medicine Hospital, Hangzhou, China

**Keywords:** acute kidney injury, chronic kidney disease, lactylation, post-translational modification, renal cell carcinoma

## Abstract

Lactylation, a novel post-translational modification (PTM) of proteins, plays a critical role in various diseases through the regulation of gene expression and metabolic reprogramming. Recent studies have increasingly highlighted its pathological and physiological relevance in kidney diseases. Lactylation exerts pleiotropic effects across diverse renal pathologies, from promoting fibrotic progression in Chronic kidney disease (CKD) to modulating immune evasion in clear cell renal cell carcinoma (ccRCC), thereby positioning it at the epicenter of metabolic-epigenetic interplay in kidney disease. This review systematically delineates the dynamic regulatory mechanisms of PTMs and their pivotal roles and current research status in kidney diseases, explores the latest advancements in lactylation research concerning CKD, acute kidney injury (AKI), and ccRCC, and discusses its potential as a therapeutic target. By synthesizing current findings, the article aims to offer new perspectives and directions for future treatments of kidney diseases and the development of targeted therapies.

## 1 Introduction

The kidney, a critical organ in maintaining systemic homeostasis, is essential for regulating endocrine functions, basal metabolism, and electrolyte balance. However, kidney diseases have become a global public health crisis, with their high prevalence and severe complications placing a significant burden on individual health, healthcare systems, and socioeconomic stability. Recent epidemiological data show that approximately 850 million people worldwide suffer from kidney diseases (Matsushita et al., 2022). CKD has become a major global public health problem. Its prevalence has steadily increased over recent decades, placing a heavy burden on health systems and economies (KDIGO, 2024). While considerable research has focused on the role of PTMs in kidney diseases, with histone acetylation and methylation well-established as drivers of AKI progression and the AKI-to-CKD transition (Guzzi et al., 2019), the emerging field of lactylation provides a fresh perspective. Notably, lactylation has been shown to directly promote renal pathological processes by modulating fibrogenic gene transcription (Zhang et al., 2024). The growing threat posed by kidney diseases is compounded by an incomplete understanding of their underlying mechanisms and a lack of therapeutic targets. Thus, further investigation into the role of lactylation in kidney diseases may identify potential therapeutic strategies and offer promising insights for the diagnosis and treatment of renal disorders.

## 2 Lactate metabolism

Since its discovery in 1780, lactate has been regarded as a metabolic end product under hypoxic conditions and a biomarker for tissue hypoxia or hypoperfusion. In the early 20th century, the Warburg effect was proposed, which asserts that cancer cells preferentially rely on glycolysis, producing significant amounts of lactate even in the presence of abundant oxygen (Fukushi et al., 2022). Subsequent research has revealed that this phenomenon of “aerobic glycolysis” extends beyond tumor cells, being observed across various normal cells and tissues, confirming that lactate is continuously produced and utilized even under oxygen-replete conditions (Li X. et al., 2022). In the 1980s, Brooks introduced the “lactate shuttle” theory, encompassing both cell-cell and intracellular lactate shuttles. This revolutionary framework shed light on the functional roles of lactate, positioning it as a critical energy carrier and signaling molecule for intercellular communication (Ferguson et al., 2018; Brooks, 2009). Lactate transport is mediated by monocarboxylate transporters (MCTs), which are widely expressed across tissues and facilitate lactate movement through passive diffusion, driven by concentration or pH gradients (Brooks, 2018; Kobayashi et al., 2021). Notably, MCT1 and MCT2 mediates lactate uptake, while MCT4 supports lactate efflux, coordinating transport and maintaining lactate homeostasis. This regulatory system underpins lactate’s role as a signaling molecule, essential for sustaining normal energy metabolism and physiological functions (Liu J. et al., 2024; Benjamin et al., 2018).

Lactic acid comprises two stereoisomers: l-lactic acid and d-lactic acid. L-lactic acid is the predominant isomer and serves as the ultimate metabolite of glycolysis. Glucose is metabolized in the cytoplasm through glycolysis to produce pyruvate (Wang et al., 2021). Under hypoxic conditions, the tricarboxylic acid (TCA) cycle is suppressed, leading to the reduction of pyruvate to lactate by lactate dehydrogenase (LDH)—a reversible redox reaction. In contrast, under aerobic conditions, lactate is converted back to pyruvate via the reverse LDH reaction. Pyruvate is then oxidized to acetyl-CoA by pyruvate dehydrogenase (PDH), entering the TCA cycle for further metabolism. D-lactic acid in humans originates mainly from gut microbiota or the methylglyoxal pathway. It is metabolized more slowly than L-lactic acid, and its accumulation in pathological states can lead to D-lactic acidosis (Drabkin et al., 2019).

Alternatively, lactate serves as a gluconeogenic precursor for glucose synthesis (Li X. et al., 2022; Adeva-Andany et al., 2014). Studies have demonstrated that the kidneys serve as a primary organ for the systemic clearance of lactic acid. This process is mediated by renal tubular cells, which facilitate active uptake, oxidation, and gluconeogenesis (Bellomo, 2002). The kidneys play a vital role in lactate metabolism, acting as a major gluconeogenic organ alongside the liver. Research indicates that during extended fasting (14–16 h), renal gluconeogenesis contributes approximately 40% of the body’s endogenous glucose production (Legouis et al., 2022). Particularly under acidic conditions, the liver’s capacity for lactic acid metabolism is impaired, whereas the kidneys exhibit a notable increase in gluconeogenic activity, underscoring their compensatory function (Bellomo, 2002). Impaired gluconeogenic capacity in renal pathologies, such as AKI and CKD, can disrupt systemic glucose homeostasis, potentially contributing to adverse clinical outcomes in affected patients (Legouis et al., 2022).

## 3 Lactylation

PTMs involve the covalent attachment of functional groups to proteins post-synthesis, influencing protein properties such as structure, activity, stability, localization, and interactions. PTMs play crucial roles in regulating various biological processes like signal transduction, gene expression, and metabolic balance. Enzymes and proteins known as “writers,” “erasers,” and “readers” precisely control the addition, removal, and recognition of PTMs, respectively (Coughlan and Testa, 2021).

In recent years, advances in proteomic technologies have accelerated research into PTMs in kidney diseases; consequently, their impact on renal pathophysiology has garnered significant attention. Various PTMs modulate distinct signaling pathways during renal injury, thereby contributing to the development of novel therapeutic strategies and the identification of potential therapeutic targets. For instance, in diabetic kidney disease (DKD), Sirtuin 1 (SIRT1) exerts protective effects by deacetylating key targets to reduce podocyte injury, inflammation, and Epithelial-Mesenchymal Transition (EMT) (Xie et al., 2024; Sun HJ. et al., 2021). In AKI, histone deacetylase 1 (HDAC1) upregulation promotes apoptosis and inflammatory responses, while HDAC inhibitors reverse these effects and ameliorate renal damage (Ranganathan et al., 2016). PTMs play crucial regulatory roles in kidney diseases through interactions among readers, writers, and erasers of PTMs. A summary in Table 1 outlines PTM types, associated kidney diseases, key targets, and effects. PTMs like lysine crotonylation and acetylation are challenging to differentiate due to shared enzymes and metabolic sensitivity. Crosstalk between SUMOylation, tyrosine modification, phosphorylation, and acetylation occurs (Li L. et al., 2024). Competitive inhibition is observed between lactoylation and acetylation, impacting p53 activation (Zong et al., 2024). Therefore, when studying lactylation, interactions with other PTMs should be carefully considered.

**TABLE 1:** Major post-translational modifications in kidney diseases.

PTM	Associated kidney diseases	Function	Target	Major biological effects	Refs
Acetylation	AKI	Pathogenic	Class IIa HDACs; SIRT2; p53	Promote inflammatory cell penetration; Inhibit cellular autophagy	Sun H. J. et al. (2021), Zhang et al. (2020), Li Y. et al. (2022), Jung et al. (2015)
		Protective	SIRT1, SIRT 3, SIRT 5, SIRT 6	Reduce apoptosis; Inhibit inflammatory signaling; Stabilize Mfn2 and protect mitochondrial function; Promotes the shift of fatty acid oxidation from mitochondria to peroxisomes	Wei et al. (2019), Shen et al. (2021), Chiba et al. (2019), Li et al. (2018)
	DKD	Pathogenic	HDAC4, HDAC5, HDAC9, BRD4	Induce podocyte injury; Aggravate inflammation and apoptosis; Promote EMT of renal tubular cells	Wang et al. (2014), Xu et al. (2021), Wang M. et al. (2024)
		Protective	SIRT1, SIRT 6	Reducing oxidative stress and activating M2 macrophages, thereby protecting podocyte function	Sun M. et al. (2021), Hong et al. (2018), Hasegawa et al. (2013), Ji et al. (2019)
	RCC	Pathogenic	BRD4, BRD9, BRDT, HDAC1, HDAC2, HDAC6, NAT10	Promotes tumor cell proliferation and migration; Transcriptionally represses KLF6	Sun et al. (2025), Wu et al. (2017), Jiang et al. (2020), Yang et al. (2014), Anraku et al. (2024)
		Protective	SIRT1	Deacetylates STAT3, suppressing tumorigenesis in RCC	Chen et al. (2019)
Glycosylation	DKD	Protective	ENTPD5	Alleviates ER stress to trigger cell proliferation in early-stage disease	Xu et al. (2023)
	IgAN	Pathogenic	IgA1	Induces mesangial cell proliferation, inflammation, and renal injury	Qi et al. (2025)
Phosphorylation	renal fibrosis from other causes	Pathogenic	FADD, SMAD2/3, PAD3, p300, p38 MAPK, EGFR	Accelerates EMT, Promotes inflammation and fibrosis	Lin et al. (2023a), Zhou et al. (2023), Li et al. (2023), Kim et al. (2025), Zeng et al. (2023), Tawengi et al. (2024)
	DKD	Pathogenic	MAP4	Induces EMT, cytoskeletal rearrangement, and apoptosis in podocytes	Li X. et al. (2022)
		Protective	AMPK	Inhibits extracellular matrix accumulation, exerting renoprotective effects	Luo et al. (2015)
	AKI	Pathogenic	PFKFB2	Impairs the glycolytic regulatory capacity of renal tubular cells, exacerbating renal fibrosis	Lee et al. (2020)
		Protective	AMPK	Inhibits ferroptosis in renal tubular cells	Du et al. (2023)
Crotonylation	renal fibrosis from other causes	Pathogenic	ACSS2	Promotes IL-1β expression, accelerating renal fibrosis	Li H. et al. (2024)
	AKI	Protective	SIRT3	Inhibits inflammatory factors	Martinez-Moreno et al. (2020)

HDAC, histone deacetylase; SIRT, sirtuin; BRD, bromodomain-containing protein; EMT, epithelial-mesenchymal transition; NAT10, N-acetyltransferase 10; KLF6, Krüppel-like factor 6; STAT3, signal transducer and activator of transcription 3; ENTPD5, ectonucleoside triphosphate diphosphohydrolase 5; ER, endoplasmic reticulum; IgA1, immunoglobulin A1; FADD, Fas-associated protein with death domain; SMAD, mothers against decapentaplegic homolog; PAD3, peptidylarginine deiminase three; p38 MAPK, p38 mitogen-activated protein kinase; EGFR, epidermal growth factor receptor; Mfn2, mitofusin 2; AMPK, AMP-activated protein kinase; PFKFB2, 6-phosphofructo-2-kinase/fructose-2, 6-bisphosphatase 2; ACSS2, acyl-CoA, synthetase short-chain family member 2; IL-1β, interleukin-1, beta.

Lactylation involves the addition of lactate groups to specific amino acid residues on proteins. This modification alters the biochemical properties and functions of the proteins (Millán-Zambrano et al., 2022). Like other PTMs such as phosphorylation, acetylation, ubiquitination, and methylation, lactylation can profoundly impact cellular signal transduction and function by regulating protein activity, stability, subcellular localization, and interactions (Wang et al., 2023). The discovery of lactylation has significantly expanded the diversity of the proteome, adding a new layer to the regulatory network linking the genome and the proteome. Currently, research on lactylation is primarily focused on two key areas: histone lactylation and non-histone lactylation. Moreover, corresponding to the two stereoisomers of lactic acid, lactylation is also divided into L-lactylation and D-lactylation.

In 2019, Professor Yingming Zhao’s team showed that glycolytic lactate can act directly as an acyl-donor precursor and, via a p300/CBP-mediated enzymatic reaction, covalently modify histones to activate transcription on chromatin (Zhang et al., 2019). This work defined lactylation as a novel histone modification and recast lactate from a metabolic end product to an epigenetic regulator. In 2020, Gaffney and colleagues reported a distinct mechanism: non-enzymatic lysine lactylation mediated by lactoyl-glutathione (Gaffney et al., 2020). This modification is enriched on glycolytic enzymes and can exert negative feedback on the glycolytic pathway, providing key evidence for the existence and regulatory importance of non-histone lactylation. In 2024, Zhang et al. combined chemical derivatization, high-performance liquid chromatography separation, and isomer-specific antibodies to identify three lactylation isomers: Lysine L-lactylation (K_l-la_), N-ε-(carboxyethyl)-lysine (K_ce_), and D-lactyl-lysine (K_d-la_). L-lactylation is the most extensively studied and functionally characterized isomer; it mainly derives from an enzymatic acyl-transfer mechanism and broadly regulates histone and non-histone protein function (Zhang et al., 2025). Therefore, unless noted otherwise, “lactylation” in this review denotes L-lactylation.

### 3.1 Enzymatic regulation of lactylation

Lactylation is dynamically regulated by specific “writers” and “erasers.” p300/CBP are widely regarded as the primary lactylation “writers.” Class I histone deacetylases (HDAC1–3) and SIRT1–3 have been shown to remove lactylation both *in vitro* and in cells, demonstrating that lactylation is reversibly regulated (Moreno-Yruela et al., 2022). In kidney disease, SIRT3 acts as an “eraser” that reduces lactylation of the mitochondrial proteins Fis1 and pyruvate dehydrogenase E1 component subunit alpha (PDHA1) at K336, thereby attenuating renal injury (An et al., 2023; Xu et al., 2026). Recent work, however, has revealed greater complexity in the lactylation catalytic network and has sparked debate within the field. Studies found that although p300 can transfer lactyl groups *in vitro*, the intracellular concentration of lactyl-CoA is much lower than that of acetyl-CoA, and—together with competitive inhibition—this disparity could substantially limit p300s lactyl-transferase activity *in vivo* (Varner et al., 2020; Ju et al., 2024). A separate study reported that p300 inhibition in macrophages does not consistently suppress lactylation, implying alternative, p300-independent lactylation pathways (Trujillo et al., 2024). In 2024, Li et al. identified alanyl-tRNA synthetase 1 (AARS1) (cytosolic) and AARS2 (mitochondrial) as lactate sensors and transferases that directly catalyze protein lactylation using lactate and ATP. This mechanism functions independently of the canonical lactyl-CoA pathway and thus defines a parallel writing route for lactylation. The study also delineated how these enzymes regulate innate immunity by lactylating the cyclic GMP-AMP synthase (cGAS), providing a mechanistic basis for lactate-mediated immunosuppression in settings such as the tumor microenvironment and infection (Li H. et al., 2024). Two studies have defined functional roles for this pathway in renal pathology. AARS1 functions as a writer that directly catalyzes histone H3 lysine 18 lactylation (H3K18-la), thereby increasing transcription of the ferroptosis-promoting gene acyl-CoA synthetase long-chain 4 (ACSL4) and promoting iron-dependent cell death in glomerular endothelial cells. H3K18-la also activates LDHA and AARS1 to form a “lactate/AARS1/H3K18-la/LDHA” positive-feedback loop that amplifies ferroptotic signaling and drives DKD progression (Hu K. et al., 2025). Hong et al. reported that AARS1 lactylates both H3K18 and STAT1, which together enhance ELOVL fatty acid elongase 5 (ELOVL5) transcription, promote lipid peroxide accumulation, and induce ferroptosis. Importantly, β-alanine inhibits AARS1 activity, blocks this pathway, suppresses ferroptosis, and improves renal function, indicating potential therapeutic utility in DKD (Hong et al., 2026). In addition, Recent studies identified GTP-specific succinyl-CoA synthetase (GTPSCS) as a nuclear lactyl-CoA synthetase that translocates from mitochondria to the nucleus and cooperates with p300 to regulate histone H3K18 lactylation (Liu R. et al., 2025). ACSS2 also functions as a lactyl-CoA synthetase that generates lactyl-CoA, and KAT2A acts as a lactyltransferase catalyzing histone H3 lactylation (Zhu et al., 2025).

### 3.2 Lactylation of histone and non-histone protein

Research on histone lactylation initially concentrated on the modification of lysine residues on histones. As essential components of nucleosomes, the basic structural units of chromatin, histones dynamically regulate chromatin structure and gene expression through lactylation. This process plays a vital role in promoting macrophage polarization from M1 to M2 during the later stages, highlighting its importance in tissue repair (Zhang et al., 2019; Irizarry-Caro et al., 2020; Wang J. et al., 2022). Histone lactylation at sites such as H3K14, H3K18, and H4K12 has been extensively studied in kidney diseases. Notably, H3K18-la exhibits pleiotropic effects across various kidney diseases. In ccRCC, H3K18-la promotes renal tumor progression through positive feedback activation of the platelet-derived growth factor receptor-β(PDGFRβ) signaling pathway (Ya et al., 2022). In AKI models, H3K18-la exacerbates tissue damage by activating inflammatory signaling pathways (Qiao et al., 2024), while in kidney stone models, increased H3K18-la levels specifically activate the transcription of fibroblast growth factor receptor 4 (FGFR4), promoting crystal deposition and renal fibrosis (Ye et al., 2025). These findings highlight the diverse regulatory roles of histone lactylation in different renal pathologies.

In contrast to the functional diversity of H3K18-la, H3K14-la and H4K12-la exhibit specific regulatory roles in renal fibrosis. H3K14-la significantly promotes EMT by upregulating Krüppel-like factor 5 (KLF5) expression (Zhang et al., 2024), while H4K12-la enhances the NF-κB signaling pathway through transcriptional regulation, thereby driving renal inflammation and fibrosis (Wang Y. et al., 2024). Investigations into the upstream regulatory mechanisms of lactylation have revealed a close association between histone lactylation and key glycolytic enzymes (including PKM2, LDHA, and PFKFB3). These glycolytic enzymes enhance glycolysis and promote lactate accumulation, thereby increasing lactylation levels. For example, galectin-3 (GAL3) increases glycolytic flux by stabilizing pyruvate kinase M2 (PKM2) (Ye et al., 2025). Moreover, FK506-binding protein 10 (FKBP10) interacts with lactate dehydrogenase A (LDHA), resulting in phosphorylation at the LDHA-Tyr10 site and promoting glycolysis (Liu R. et al., 2024). Additionally, Wang et al. demonstrated that upregulation of 6-phosphofructo-2-kinase/fructose-2,6-bisphosphatase 3 (PFKFB3) stimulates glycolysis and increases lactylation (Wang Y. et al., 2024). However, the upstream mechanism regulating PFKFB3 remains unclear, and elucidating this mechanism could be a promising avenue for future research (Wang Y. et al., 2024).

Gaffney et al. reported lactylation of non-histone proteins. In 2022, Wan et al. (2022) utilized proteomic data analysis to validate the sensitivity and specificity of cyclic immonium (CycIm) ions for identifying protein lactylation modifications. The study highlighted lactylation as a widespread post-translational modification occurring not only on histones but also extensively on non-histone proteins, particularly enriched in glycolytic enzymes, thereby providing a theoretical foundation for subsequent research on non-histone lactylation. Furthermore, this work established a reliable identification strategy for lactylation, which is expected to further advance functional studies on lactylation in physiological and pathological contexts (Wan et al., 2022). In 2023, Gao et al. (Yang Z. et al., 2023) identified a total of 9,275 lysine lactylation (Kla) sites, with over 99% located on non-histone proteins, further confirming lactylation as a prevalent modification extending beyond histones and broadly involved in diverse cellular biological processes. These findings offer new insights for exploring the link between non-histone lactylation and disease mechanisms. For instance, studies have shown that lactylation of the transcription factor c-Myc promotes tubular epithelial cell proliferation and accelerates recovery after ischemia-reperfusion injury (Li Y. et al., 2024); in sepsis-induced acute kidney injury (S-AKI), lactylation at the K20 site of mitochondrial fission protein Fis1 has been demonstrated to drive aberrant mitochondrial fission and exacerbate cellular apoptosis (An et al., 2023); moreover, lactylation at the K52 site of aldehyde dehydrogenase 2 (ALDH2) aggravates tubular injury and mitochondrial dysfunction, while SIRT3-mediated delactylation exhibits protective effects (Li et al., 2025).

As research progresses, scientists have increasingly recognized the critical roles of lactylation in various physiological and pathological processes (Yang et al., 2022; Wa et al., 2023). These findings significantly expand our understanding of lactylation’s biological significance, extending beyond its initial characterization as a histone modification. The discovery of non-histone lactylation has opened new research avenues for metabolic regulation and disease mechanisms. Furthermore, non-enzymatic factors, including specific gene expression and neuronal excitation states, have been shown to modulate lactylation levels (Hagihara et al., 2021). This highlights the complexity and diversity of lactylation, highlighting its essential roles in cellular signaling and biological functions. As a novel PTM, the precise enzymatic mechanisms, tissue-specific regulatory patterns, and roles of lactylation in disease pathogenesis remain to be fully elucidated through future investigations.

**FIGURE 1:**
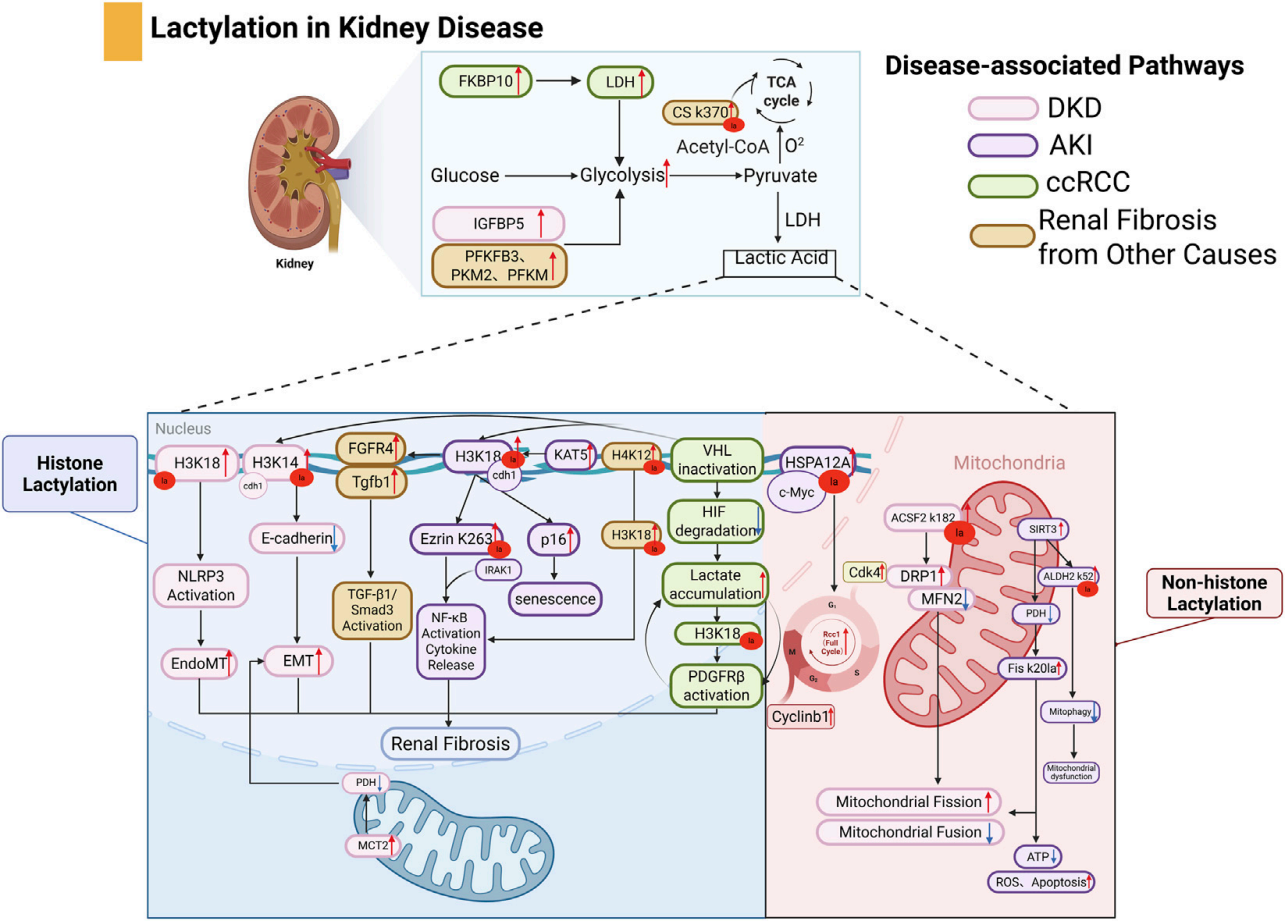
Roles of histone (left) and non-histone (right) lactylation in kidney diseases. FKBP10, FK506-binding protein 10; IGFBP5, Insulin-like growth factor binding protein 5; PFKFB3: 6-phosphofructo-2-kinase/fructose-2,6-bisphosphatase 3; PKM2, pyruvate kinase M2; H3K14: Histone H3 lysine 14; H3K18, Histone H3 lysine 18; FGFR4: fibroblast growth factor receptor 4; NLRP3, NLR family pyrin domain containing 3; TGF-β1/Smad3, Transforming growth factor beta 1/Smad family member; EMT, Epithelial-mesenchymal transition; Ezrin K263la: Ezrin lactylation at lysine 263; VHL, Von Hippel-Lindau tumor suppressor; PDGFRβ, Platelet-derived growth factor receptor beta; HSPA12A, Heat shock protein A12A; C-Myc, Cellular myelocytomatosis oncogene; Cdk4, Cyclin-dependent kinase 4; MCT2: monocarboxylate transporter 2; FKBP10: FK506-binding protein 10; MFN2, mitofusin 2; DRP1, dynamin-related protein 1; SIRT3, sirtuin 3; ALDH2 K52, aldehyde dehydrogenase 2 lysine 52; PDH, pyruvate dehydrogenase complex; Fis1 K20, mitochondrial fission 1 protein lysine 20; ACSF2 K182, acyl-CoA synthetase family member 2 lysine 182; PFKM, phosphofructokinase muscle type; CS: Citrate Synthase.

In summary, lactylation is a metabolite-driven, dynamically reversible post-translational modification that contributes to renal disease pathogenesis via a complex enzymatic regulatory network, dynamic modulation of transcription at key histone loci, and site-specific modification of non-histone proteins (Figure 1). Table 2 summarizes representative lactylation sites and their functional characterization in renal diseases reported in recent years.

**TABLE 2:** Role of lactylation in kidney disease: implications and future perspectives.

Studies	Diseases	Lactylation category	Lactylation proteins	Implications	Directions for future research	Refs
Zhang et al. (2024)	DKD	histone lactylation	H3K14	KLF5 inhibition may serve as a potential therapeutic strategy for DKD	Elucidating the functional impact of H3K14-la mutations on KLF5 and the crosstalk between histone lactylation and acetylation in regulating gene expression in renal tubular epithelial cells	Zhang et al. (2024)
Chen et al. (2024a)	DKD	Non-histone lactylation	ACSF2 K182	Targeting mitochondrial ACSF2 and its lactylation may serve as a potential therapeutic approach for DKD.	Investigating the specific mechanisms by which lysine lactylation at the K182 site of ACSF2 influences DKD progression, or developing targeted intervention drugs/therapeutic strategies	Chen et al. (2024a)
Zheng et al. (2025)	DKD	histone lactylation	(Not specified)	Targeting histone lactylation or its upstream regulators may enable early intervention for renal fibrotic diseases	Clarifying the mechanisms underlying lactate accumulation in podocytes under chronic hyperglycemic conditions	Zheng et al. (2025)
Chen et al. (2025)	DKD	histone lactylation	H3K18 as the main target	Glis1 may serve as a therapeutic target by inhibiting KAT5-mediated lactylation	Further investigate the specific role and mechanism of lactylation in DKD, clarify the regulatory mechanism of Glis1 on KAT5, and how this interaction affects the development of DKD.	Chen et al. (2025)
Hu X. et al. (2025)	DKD	histone lactylation	H3K18	IGFBP5 promotes EndoMT and renal fibrosis by regulating histone H3K18-la, elucidating a novel molecular pathway in DKD progression	Future studies should investigate other IGFBP5-related regulatory pathways and interactions to elucidate its role in DKD progression and validate its therapeutic potential in humans	Hu X. et al. (2025)
Hong et al. (2026)	DKD	Histone lactylation and Non-histone lactylation	H3K18 and STAT1 K193	Targeting AARS1-mediated H3K18 and STAT1 lactylation to suppress ELOVL5 transcription and ferroptosis may treat DKD.	Investigate the therapeutic effects of β-alanine as a competitive inhibitor of AARS1 in DKD animal models; Expand clinical sample size to further analyze the correlation between AARS1/H3K18-la and disease progression	Hong et al. (2026)
Hu K. et al. (2025)	DKD	Histone lactylation	H3K18	The lactate/AARS1/H3K18-la/LDHA feedback loop drives ferroptosis via ACSL4 in DKD. Blocking this loop with β-alanine confers protection, suggesting potential therapeutic strategies for DKD	Investigate ACSL4-driven lipid metabolism in DKD ferroptosis; assess β-alanine efficacy and safety in DKD; explore this pathway in other diabetic complications.	Hu K. et al. (2025)
An et al. (2023)	SAKI	Non-histone lactylation	Fis1 K20	Reducing lactate levels and Fis1 lactylation may alleviate S-AKI	Elucidation of the mechanisms controlling Fis1 K20 lactylation	An et al. (2023)
Li et al. (2025)	SAKI	Non-histone lactylation	ALDH2 K52	Modulating ALDH2 lactylation or enhancing mitophagy may treat AKI	The role of other Sirtuin family proteins in de-lactylation remains to be evaluated, and the interaction between acetylation and lactylation also needs further investigation	Li et al. (2025)
Wei et al. (2025)	SAKI	histone lactylation	HMGB1	Targeting macrophage HMGB1 lactylation may treat S-AKI	To investigate HMGB1’s interaction partners, downstream signaling, and other factors regulating NET formation and HMGB1 function in SAKI.	Wei et al. (2025)
Qiao J et al. (2024)	SAKI	histone lactylation	H3K18, Ezrin K263	Inhibiting H3K18-la may mitigate S-AKI	Delving into the regulatory mechanisms of histone and Ezrin lactylation and exploring associated signaling pathways and targets	Qiao et al. (2024)
Li Y. et al. (2024)	IRI-AKI	Non-histone lactylation	c-Myc	Targeting HSPA12A may enhance renal recovery post-IRI	Investigating whether HSPA12A also participates in renal fibrosis or post-chronic injury repair via this mechanism in other kidney diseases	Li Y. et al. (2024)
Zhou et al. (2024)	IRI-AKI	histone lactylation	H3K18	AST-120 may interrupt this HK2-glycolysis-lactate-H3K18 lactylation positive feedback loop by inhibiting HK2-mediated glycolysis	Elucidating the specific upstream regulatory mechanisms by which AST-120 downregulates HK2 expression	Zhou et al. (2024)
Yang et al. (2022)	ccRCC	histone lactylation	H3K18 as the main target	Inhibiting histone lactylation and PDGFRβsignaling as ccRCC therapy	Further dissecting the heterogeneous role of histone lactylation in the tumor microenvironment of ccRCC.	Ya et al. (2022)
Yang et al. (2023b)	ccRCC	histone lactylation	(Not specified)	HIBCH was identified as a potential biomarker and therapeutic target in ccRCC.	Investigating the specific molecular mechanisms of HIBCH in ccRCC and its interaction with the immune microenvironment	Yang et al. (2023b)
Liu J. et al. (2024)	ccRCC	histone lactylation	LDHA Y10	Elucidating the critical role of FKBP10 in ccRCC progression and its potential as an independent prognostic biomarker for ccRCC.	Conducting clinical studies on the correlation between FKBP10 expression and HIF2 inhibitor efficacy, and validating its value as a predictive biomarker for treatment response	Liu J. et al. (2024)
Ye et al. (2025)	kidney stones	histone lactylation	H3K18	Modulation of histone lactylation or FGFR4 signaling may offer new treatment strategies	Investigating the structural basis of Lgals3-PKM2 interaction and analyzing the role of Lgals3 across renal cell subpopulations	Ye et al. (2025)
Xiang et al. (2025)	Renal Fibrosis from Other Causes	histone lactylation	H3K18	Shikonin-mediated PKM2 inhibition attenuates lactylation and fibrosis, suggesting therapeutic potential	Defining the cell-specific role of PKM2 in renal fibrosis	Xiang et al. (2025)
Wang M. et al. (2024)	Renal Fibrosis from Other Causes	histone lactylation	H4K12	Targeting PFKFB3 and lactylation-mediated NF-κB signaling may be a new treatment for kidney fibrosis	Dissecting the writers/erasers of H4K12-la and elucidating the impact of non-histone lactylation on NF-κB activation	Wang Y. et al. (2024)
Chen et al. (2026)	Renal Fibrosis from Other Causes	histone lactylation	H3K18	Targeting the PFKM/glycolysis/lactate/H3K18-la/NF-κB axis to inhibit renal fibrosis may treat CKD.	Validate the role of PFKM in various renal fibrosis models and clarify its specificity in further animal studies	Chen et al. (2026)
Tang et al. (2026)	Renal Fibrosis from Other Causes	histone lactylation	H4K12	TMAO modulates renal tubular metabolism via the gut–kidney axis and drives M2 polarization via H4K12-la, with p300 playing a key role in this process	Validate the TMAO-CKD progression link in larger clinical cohorts; examine TMAO effects on lipid and overall energy metabolism	Tang et al. (2026)
Yu et al. (2025a)	Renal Fibrosis from Other Causes	Non-histone lactylation	CS K370	CS K370-la promotes AKI-to-CKD transition via CS inactivation and NLRP3 activation, indicating CS lactylation as a key regulatory target for this process	Elucidate the molecular mechanism of CS lactification on enzyme activity; examine the role of metabolic enzyme lactylation in other pathways during AKI-CKD transition	Yu et al. (2025a)
Xu et al. (2026)	IgAN	Non-histone lactylation	PDHA1 K336	PDHA1 lactylation drives B cell metabolic reprogramming in IgAN, and telitacicept acts via the SIRT3-PDHA1 pathway to regulate B cell metabolism	Further explore the regulatory mechanism between BAFF/April signaling and SIRT3 expression	Xu et al. (2026)

KLF5, Krüppel-like factor 5; ACSF2, acyl-CoA, synthetase family member 2; Glis1, GLIS, family zinc finger 1; KAT5, lysine acetyltransferase 5; IGFBP5, insulin-like growth factor binding protein 5; EndoMT, endothelial-mesenchymal transition; Fis1, mitochondrial fission 1 protein; ALDH2, aldehyde dehydrogenase 2; HMGB1, high mobility group box 1; NET, neutrophil extracellular trap; HSPA12A, heat shock protein A12A; HK2, hexokinase 2; PDGFRβ, platelet-derived growth factor receptor-β; HIBCH, 3-hydroxyisobutyryl-CoA, hydrolase; FKBP10, FK506-binding protein 10; LDHA, lactate dehydrogenase A; HIF2α, hypoxia-inducible factor 2α; FGFR4, fibroblast growth factor receptor 4; Lgals3, galectin-3; PKM2, pyruvate kinase M2; PFKFB3, 6-phosphofructo-2-kinase/fructose-2, 6-bisphosphatase 3; NF-κB, nuclear factor kappa-B; H3K14, histone H3 lysine 14; H3K18, histone H3 lysine 18; H4K12, histone H4 lysine 12; AARS1, alanyl-tRNA, synthetase 1; NLRP3, NLR, family pyrin domain containing 3; ELOVL5, ELOVL, fatty acid elongase 5; ACSL4, acyl-CoA, synthetase long-chain 4; PFKM, phosphofructokinase muscle type; TMAO, trimethylamine N-oxide; CS, citrate synthase; PDHA1, pyruvate dehydrogenase E1 component subunit alpha.

## 4 Lactylation and kidney diseases

### 4.1 CKD-related disorders

CKD progression culminates in renal fibrosis, a pathological state driven by interacting mechanisms such as chronic inflammation, phenotypic conversion of intrinsic renal cells, mitochondrial dysfunction, and metabolic reprogramming (Miguel and Kramann, 2023; Panizo et al., 2021). Recent studies indicate that increased lactate production and protein lactylation are important drivers of renal fibrosis.

Among the causes of CKD, DKD is the most illustrative model for studying lactylation because of its high prevalence and strong links to metabolic dysfunction. DKD also ranks among the most severe microvascular complications of diabetes, affecting approximately 40% of people with diabetes worldwide. It has become a major contributor to the global disease burden and the leading cause of end-stage kidney disease (Alicic et al., 2017; Lin et al., 2023b). Poor glycemic control is a modifiable risk factor for the development and progression of DKD, a finding consistently supported by subsequent research (Alicic et al., 2017). Hyperglycemia in diabetes promotes accumulation of both L-lactate and D-lactate in the kidney. Several studies report abnormally elevated D-lactate levels in diabetic patients, suggesting D-lactate as a potential circulating biomarker for diabetic vascular complications, including DKD (Scheijen et al., 2012). Hyperglycemic conditions lead to lactate accumulation through dual mechanisms: upregulation of MCT2 expression and inhibition of PDH activity, subsequently increasing histone lactylation modifications. Histone lysine lactylation has been found to directly activate podocyte EMT and disrupt the glomerular filtration barrier. Inhibition of lactate production has been shown to reduce histone lysine lactylation levels, reverse EMT, and improve renal function (Zheng et al., 2025). Zhang et al. demonstrated that H3K14-la enhances KLF5 expression, which then binds to the E-cadherin promoter region, repressing its transcriptional activity. This epigenetic regulation leads to downregulation of E-cadherin, promoting EMT and renal fibrosis. Both genetic and pharmacological inhibition of KLF5 have been demonstrated to effectively attenuate these pathological processes (Zhang et al., 2024). Recent studies have also revealed that insulin-like growth factor binding protein 5 (IGFBP5) is upregulated under hyperglycemic conditions, promoting lactate production by enhancing glycolytic flux. This, in turn, elevates histone H3K18 lactylation levels. This modification activates the NLR family pyrin domain containing 3 (NLRP3) inflammasome, which drives EMT and fibrotic progression. Genetic knockdown of IGFBP5 has been shown to significantly inhibit this pathway, thereby alleviating renal injury (Hu X. et al., 2025). The hyperglycemic environment in diabetes can lead to renal lactate accumulation, which, in turn, induces intracellular mitochondrial dysfunction—an essential factor in the pathogenesis of DKD (Chen J. et al., 2024). The kidney, a high–energy–consuming organ, depends on mitochondria to carry out critical functions and maintain systemic homeostasis. Beyond ATP production, mitochondria respond to metabolic shifts through integrated signaling pathways (Bhargava and Schnellmann, 2017). Accumulated lactic acid strongly inhibits mitochondrial oxidative phosphorylation (OXPHOS) and mitophagy, lowers intracellular pH, suppresses electron transport chain (ETC.) activity, and reduces ATP production. Excess lactic acid also directly inhibits NADH dehydrogenase, worsening mitochondrial dysfunction. As mitochondrial damage decreases ATP synthesis, cells shift toward glycolysis, which increases lactic acid production and creates a vicious cycle of glycolysis dependence and lactic acid accumulation (Darshi et al., 2024; Huang et al., 2025). Lactic acid levels also influence mitochondrial dynamics; maintaining mitochondrial homeostasis through balanced fission and fusion is essential. Mitochondria fusion is mediated by the GTPases Opa1 and Mitofusin-1 (Mfn1) and Mfn2, whereas fission is mediated by the mitochondrial fission one protein (Fis1), mitochondrial fission factor (Mff), and dynamin-related protein (Drp1), which respond to metabolic cues such as lactic acid to enable adaptive remodeling of mitochondrial morphology and function (Khan et al., 2023; Zanfardino et al., 2025). In DKD, Chen et al. conducted comprehensive lactylome analyses, identifying elevated lactylation at 356 sites across 165 proteins in mouse kidneys, with predominant mitochondrial localization. Through a series of cellular experiments, they demonstrated that acyl-CoA synthetase short-chain family member 2 (ACSF2) predominantly localizes to mitochondria, and its lactylation at the K182 site disrupts mitochondrial homeostasis, leading to mitochondrial dysfunction. This molecular alteration ultimately results in renal tubular epithelial cell (RTEC) injury and may contribute to DKD progression (Chen J. et al., 2024).

In addition to driving fibrosis and mitochondrial dysfunction, lactylation also participates in the regulation of senescence in renal tubular epithelial cells. GLIS family zinc finger 1 (GLIS1) acts as a novel endogenous regulator of RTEC senescence. This research demonstrated that GLIS1 competitively binds to lysine acetyltransferase 5 (KAT5), inhibiting the formation of H3K18-la and consequently delaying p16-mediated cellular senescence in RTECs (Chen et al., 2025). This discovery provides valuable insights into the complex regulation of cellular aging in diabetic nephropathy.

Lactylation, a convergent pathway that promotes renal fibrosis, is not limited to diabetic kidney disease and likewise conforms to a core “metabolism–epigenetics–inflammation” axis. In CKD of various etiologies, dysregulated glycolytic flux and the resulting lactate accumulation act as an upstream hub activating pro-fibrotic signaling. For example, PFKFB3 is markedly upregulated in fibrotic renal tissue and promotes histone H4K12la, thereby activating the NF-κB family and driving kidney inflammation and fibrosis (Wang Y. et al., 2024). Likewise, the glycolytic enzyme PFKM activates NF-κB through lactate-mediated H3K18 lactylation, which advances renal fibrotic progression (Chen et al., 2026). PKM2 expression is increased and lactate production rises, which promotes histone H3K18 lactylation and thereby induces TGF-β1 expression, driving macrophage-to-myofibroblast transition (MMT).

Based on this mechanism, investigators have proposed therapeutic strategies: shikonin reduces lactate production by inhibiting PKM2 activity, disrupting the TGF-β1/Smad3 pathway and attenuating renal fibrosis progression (Xiang et al., 2025). Building on this metabolic–epigenetic regulatory axis, researchers have tested additional interventions. Gentisic acid inhibits key glycolytic enzymes, lowers lactate generation and H3K18-la levels, and effectively suppresses renal fibrosis (Wang et al., 2026). In remodeling the immune microenvironment, The gut-derived metabolite trimethylamine N-oxide (TMAO) induces H4K12la, which drives macrophage polarization toward the M2 phenotype and thereby accelerates CKD pathology (Tang et al., 2026). This mechanism connects the gut–kidney axis to epigenetic modification, implying that altering gut microbiota–derived metabolites or directly targeting H4K12la could improve the immune microenvironment in CKD. Moreover, during the pivotal conversion from AKI-to-CKD conversion, lactylation of citrate synthase activates the NLRP3 inflammasome and promotes maturation and release of proinflammatory cytokines such as IL-1β and IL-18. This sustained inflammatory milieu impairs normal repair, activates fibroblasts, and enhances extracellular matrix deposition, ultimately causing irreversible renal fibrosis and facilitating progression from AKI to CKD (Yu F. et al., 2025). This central axis also plays a critical role in immune-mediated CKD. Studies show that B cells undergo pronounced metabolic reprogramming after activation. Xu et al. reported increased lactylation at PDHA1 K336 in B cells from patients with IgA nephropathy (IgAN). This modification inhibits PDH activity, thereby promoting glycolysis, lactate production, and IgA1 secretion, which contribute to renal injury. Telitacicept upregulates the mitochondrial deacetylase SIRT3 and promotes delactylation of PDHA1 at K336, restoring PDH activity and OXPHOS, reversing the shift of B cells toward glycolysis, and reducing lactate production and Gd-IgA1 secretion. In addition, Xu’s group used co-culture experiments to show that reduced lactate directly attenuates lactate-driven inflammation (IL-6/TNF-α upregulation), fibrosis (α-SMA/Col I overexpression), and apoptosis in human mesangial cells (HMCs), thereby blocking progression of IgAN-related renal lesions (Xu et al., 2026).

### 4.2 Acute kidney injury (AKI)

AKI is a critical clinical syndrome characterized by a rapid decline in renal function and the accumulation of metabolic waste, among which S-AKI is the most common and is consistently associated with elevated mortality rates and unfavorable clinical outcomes (Ferenbach and Bonventre, 2015; Peerapornratana et al., 2019). The pathogenic mechanisms of AKI primarily include hypoxia, ischemia-reperfusion injury, inflammatory responses, and nephrotoxic effects of drugs (An et al., 2023; Corridon, 2023; Perazella and Rosner, 2022). Multiple clinical studies have shown that elevated lactate levels correlate positively with both the incidence and severity of S-AKI, and that lactate is an independent risk factor for worse outcomes in S-AKI (An et al., 2023; Kim et al., 2023). An et al. further demonstrated that lactate-mediated protein lactylation is a key mechanism driving kidney injury. Their work indicates that non-histone protein lactylation plays a pivotal role in mitochondrial dysfunction during the pathogenesis of S-AKI. In the development of sepsis-induced AKI, downregulation of the deacetylase SIRT3 leads to hyperacetylation of pyruvate dehydrogenase E1 component subunit alpha (PDHA1), inhibiting its enzymatic activity. This metabolic shift promotes excessive conversion of pyruvate to lactate. The accumulated lactate subsequently induces lactylation of mitochondrial fission one protein at K20 (FIS1 K20la), driving pathological mitochondrial fragmentation. These alterations result in ATP depletion, excessive ROS accumulation, and ultimately, cellular apoptosis, exacerbating S-AKI progression (An et al., 2023). Notably, SIRT3 exhibits dual enzymatic functions, acting as both a deacetylase and a delactylase for aldehyde dehydrogenase 2 (ALDH2) (Li et al., 2025). In both patients with AKI and murine models, renal tissues demonstrate significantly elevated lactate levels and pan-histone lactylation (Kla). Single-cell RNA sequencing has revealed pronounced lactylation activity in injured proximal tubular cells, with significant elevation of ALDH2 lactylation at K52. This modification exacerbates tubular damage and mitochondrial dysfunction. In contrast, the ALDH2/K52R mutation alleviates these pathological changes in HK-2 cells and kidney tissues. Moreover, upregulation of SIRT3 reduces ALDH2 lactylation, improving tubular injury and mitochondrial dysfunction (Li et al., 2025). These findings not only shed light on the molecular mechanisms of lactylation in S-AKI pathogenesis but also lay the groundwork for therapeutic strategies targeting the SIRT3-ALDH2 axis.

Increasing evidence demonstrates that lactylation exacerbates S-AKI progression through pro-inflammatory mechanisms. Research indicates that lactate enhances inflammation via two distinct pathways: First, lactate stimulates M1 macrophages to secrete exosomes that promote the cytoplasmic accumulation and release of high mobility group box 1 (HMGB1). Concurrently, lactate induces HMGB1 lactylation. Lactylated HMGB1 activates cGAS/stimulator of interferon genes (STING) signaling pathway, leading to aberrant release of mitochondrial DNA (mtDNA) from neutrophils. The extracellular mtDNA acts as damage-associated molecular patterns (DAMPs), facilitating neutrophil extracellular trap (NET) formation, which activates immune responses and amplifies inflammation, further aggravating S-AKI (Wei et al., 2025). Second, in S-AKI models, elevated renal lactate levels significantly increase H3K18-la. This modification specifically enriches at the RhoA gene promoter region, activating the RhoA-ROCK1-Ezrin signaling cascade. Notably, lactylation of Ezrin at K263 enhances its interaction with interleukin-1 receptor-associated kinase 1 (IRAK1), triggering NF-κB activation and pro-inflammatory cytokine release, ultimately exacerbating renal injury (Qiao et al., 2024).

Ischemia-reperfusion injury (IRI) is a major contributor to AKI (Wang S. et al., 2022). The kidney, as one of the most highly vascularized organs, is particularly susceptible to ischemic damage (Lafrance and Miller, 2010). During IRI, reduced blood flow induces renal tissue hypoxia, triggering renal cells to enhance glycolytic flux to produce ATP and maintain energy balance. Hexokinase 2 (HK2), a key rate-limiting enzyme in glycolysis, plays a critical role in this adaptive metabolic response. While the upregulation of glycolysis may transiently alleviate acute stress-induced renal injury in the early stages of AKI, prolonged glycolytic activation ultimately exacerbates renal damage (Li et al., 2021). Following IRI, HK2-driven glycolysis increases intracellular lactate levels, which in turn promotes H3K18-la. H3K18-la accumulates at the HK2 promoter region, further enhancing HK2 transcription and creating a feedforward loop that sustains glycolytic hyperactivity. Notably, the spherical carbon adsorbent AST-120 disrupts this pathological cycle by suppressing HK2 expression, reducing glycolysis, and lowering H3K18 lactylation levels. By targeting this metabolic-epigenetic axis, AST-120 mitigates IRI-induced kidney injury and promotes functional recovery in AKI (Zhou et al., 2024). Following AKI, renal tubules exhibit differential repair responses, with some undergoing atrophy while others fully recover (Venkatachalam et al., 2015). This heterogeneity in repair capacity plays a critical role in the progression from AKI to CKD (Moradi and Wang, 2013). Experimental data confirm that the proliferative capacity of RTECs is the key determinant of tubular structural remodeling and functional recovery (Chang-Panesso et al., 2019). The transcription factor c-Myc has been shown to significantly promote TEC proliferation after IRI (Patel et al., 2004). Recent mechanistic studies revealed that heat shock protein A12A (HSPA12A) directly interacts with c-Myc and upregulates the hypoxia-inducible factor 1α (HIF1α)-dependent glycolytic pathway, enhancing lactate production. This metabolic shift increases c-Myc lactylation, promoting its nuclear translocation and subsequent activation of pro-proliferative gene expression programs, which drive TEC proliferation (Li Y. et al., 2024). This study identified the HSPA12A/c-Myc signaling axis as a novel regulator of tubular repair via lactylation-mediated epigenetic modulation, providing a rationale for developing small-molecule HSPA12A agonists as a promising strategy to enhance renal functional recovery after IRI.

### 4.3 Renal cell carcinoma (RCC)

RCC, a prevalent malignancy of the urinary system, predominantly originates from RTECs. According to the 2022 National Comprehensive Cancer Network (NCCN) Guidelines for Kidney Cancer, RCC accounts for approximately 90% of all renal malignancies, with ccRCC being the most prevalent and clinically significant subtype (Motzer et al., 2022). Most ccRCC cases harbor loss-of-function mutations in the Von Hippel-Lindau (VHL) tumor suppressor gene (Ya et al., 2022). Under normal conditions, VHL targets hypoxia-inducible factors (HIFs) for ubiquitin-mediated degradation. Loss of VHL function stabilizes HIF-α subunits, notably HIF-1α and HIF-2α, under normoxia and thereby establishes a “pseudo-hypoxic” state (Liu Y. et al., 2025). This central genetic lesion drives extensive metabolic reprogramming in cancer cells. Sustained HIF activation shifts metabolism toward aerobic glycolysis despite adequate oxygen, producing large amounts of lactic acid (the Warburg effect). This shift both supplies energy and biosynthetic precursors for rapid proliferation and leads to pronounced lactic acid accumulation within tumor cells and the surrounding microenvironment (Sanchez and Simon, 2018).

This metabolic phenotype not only reflects altered energy metabolism but also functions as an epigenetic conduit that promotes tumor progression and immune evasion via histone lactylation. Loss-of-function mutations in the VHL tumor suppressor activate PDGFRβ signaling through histone lactylation, creating a self-perpetuating loop: “VHL deficiency—lactate accumulation—histone lactylation—PDGFRβ hyperactivation.” Both *in vitro* and *in vivo* studies show that disrupting this cascade, for example, with lactylation inhibitors, markedly reduces ccRCC cell proliferation, invasion, and metastatic capacity (Ya et al., 2022). These findings suggest that targeting lactylation-dependent regulatory nodes could offer a new therapeutic avenue for ccRCC. Lactylation also remodels the tumor microenvironment (TME) toward immunosuppression by modulating immune-related gene expression. Accumulated lactate in the TME increases expression of the m6A reader YTH N6-methyladenosine RNA binding protein 2 (YTHDF2) in tumor cells via histone lactylation, promoting degradation of tumor suppressor transcripts and thus driving tumorigenesis. Lactate likewise elevates the m6A methyltransferase Methyltransferase-like 3 (METTL3) in tumor-infiltrating myeloid cells (TIMs), which enhances their immunosuppressive activity through m6A-dependent mechanisms and facilitates immune escape (Yu et al., 2021; Xiong et al., 2022). In the study of ccRCC, By integrating m6A methylation-related genes, the lactylation-regulated metabolic-epigenetic network was found to significantly influence the immune-suppressive characteristics of the tumor microenvironment (TME). Key lactylation-related genes, such as 3-hydroxyisobutyryl-CoA hydrolase (HIBCH), were identified, with their expression levels significantly correlating with patient prognosis: Low HIBCH expression is linked not only to mitochondrial dysfunction but also to upregulation of immune checkpoints like CTLA-4 and PD-1, promoting immune escape (Yang L. et al., 2023). Similarly, the RNA methyltransferase NSUN2 stabilizes NEO1 mRNA, thereby enhancing glycolysis. The resulting lactate accumulation drives H3K18-la, which in turn activates a transcriptional program involving MYC and POM121 to upregulate the expression of the immune checkpoint PD-L1 (encoded by CD274), further facilitating tumor immune evasion (Wang et al., 2025). Prognostic models incorporating lactylation-related gene signatures, such as the HIBCH model for m6A and Mitoscore model for m5C, effectively predict immunotherapy response, offering novel tools for clinical decision-making (Yang L. et al., 2023; Wang et al., 2025). In addition, FKBP10 binds LDHA via its C-terminal domain and promotes phosphorylation at tyrosine 10 (Y10), which enhances glycolytic activity. This interaction markedly increases tumor-cell glycolysis and lactate production and thereby drives ccRCC invasion and metastasis through histone lactylation. Together, these findings reveal a novel mechanism in which coordinated PTMs (phosphorylation and lactylation) act synergistically to promote disease progression and open new avenues for research in renal malignancies (Liu R. et al., 2024).

### 4.4 Other kidney contexts

Kidney transplantation is the most effective treatment for patients with end-stage kidney disease. Long-term graft survival, however, remains problematic. Chronic allograft dysfunction, histologically characterized by interstitial fibrosis and tubular atrophy, is detectable in 25% of transplanted kidneys within 1 year after transplantation (Heilman et al., 2015). Although calcineurin inhibitors have greatly reduced acute rejection, ischemia–reperfusion injury and immune-mediated chronic rejection continue to drive progressive allograft fibrosis (Li and Zhuang, 2014). Recent work implicates lactylation in driving renal allograft fibrosis. Single-nucleus RNA sequencing of biopsy samples showed a marked increase in the lactylation score in fibrotic tissue. The analysis identified five putative driver genes—IKZF1, PDLIM1, S100A11, STAT4, and SLC2A3—and localized their expression predominantly to immune and stromal cell populations. Upregulation of these genes promotes interstitial fibrosis and tubular atrophy by reshaping the immune microenvironment and enhancing inflammatory activation (Yuan et al., 2026). Molecular studies have shown that nucleophosmin 1 (NPM1) is lactylated in the transplanted kidney. This lactate–NPM1 lactylation axis mediates ferroptotic waves that propagate ferroptosis signals and cellular damage, thereby aggravating early graft dysfunction (Yu H. et al., 2025). Lactylation also contributes to the pathogenesis of other kidney diseases beyond transplanted kidney fibrosis. In kidney stones, galectin-3 (Lgals3) binds directly to the glycolytic enzyme PKM2 and prevents its Trim21-mediated ubiquitination and degradation, thereby stabilizing PKM2 and accelerating glycolysis and lactate production. The resulting lactate accumulation increases H3K18 lactylation, which is enriched at the FGFR4 promoter and promotes FGFR4 transcription. Elevated FGFR4 signaling then aggravates renal fibrosis and crystal deposition (Ye et al., 2025). These findings highlight a critical role for lactylation in kidney stone formation and secondary renal fibrosis, expanding the recognized pathological scope of lactylation in kidney disease.

## 5 Summary and perspectives

**FIGURE 2:**
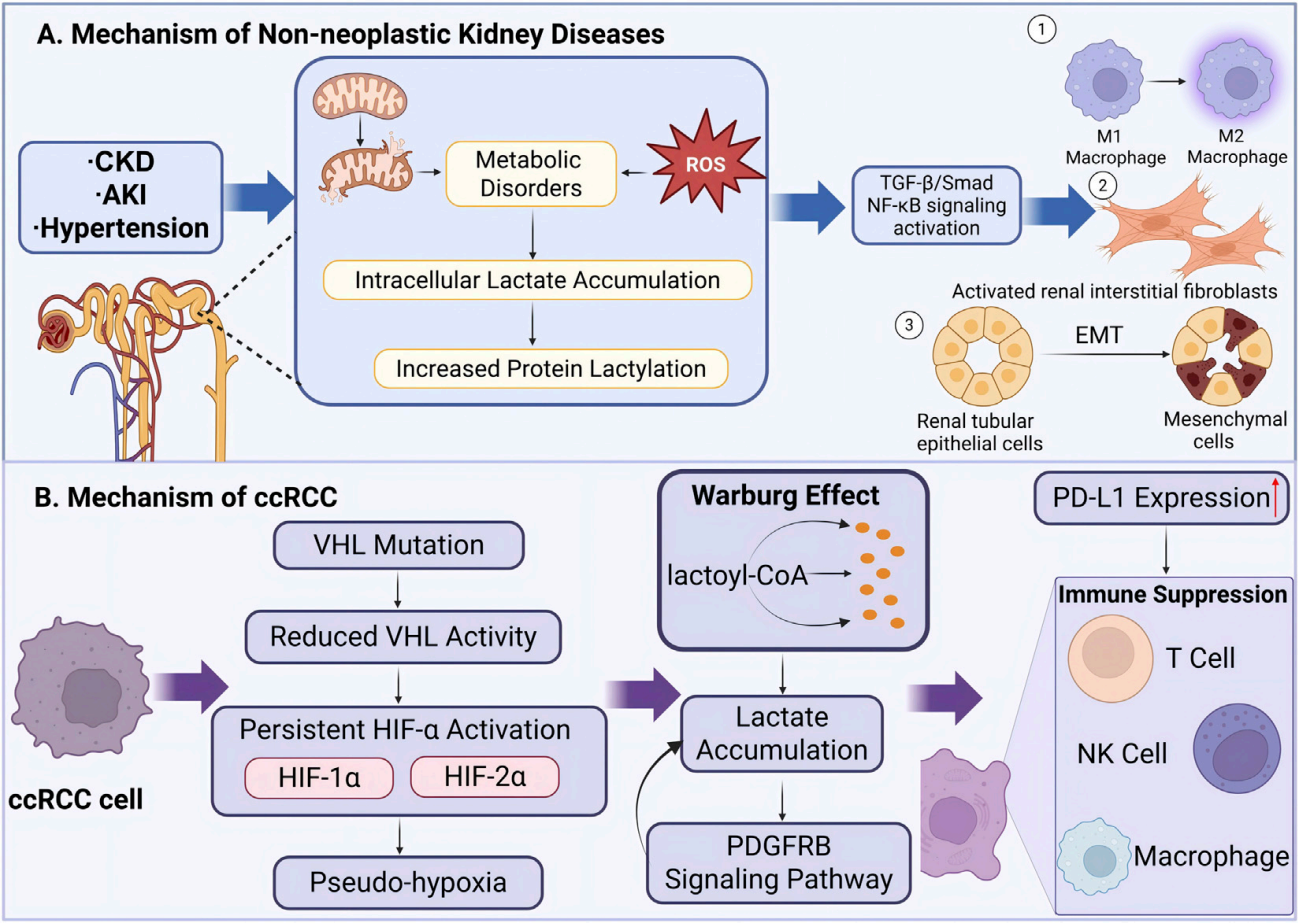
Divergent mechanisms of lactylation in non-neoplastic kidney diseases **(A)** and clear cell renal cell carcinoma **(B)** ROS, reactive oxygen species; EMT, epithelial-mesenchymal transition; VHL, von Hippel-Lindau; HIF-α, hypoxia-inducible factor α; PDGFRβ, platelet-derived growth factor receptor-β; NK cell, Natural killer cell.

Lactylation serves as a critical link between cellular metabolism and epigenetic regulation, establishing a sophisticated and complex regulatory network in kidney diseases. This review systematically summarizes the mechanistic roles of lactylation in diabetic nephropathy, AKI, and clear cell renal carcinoma through its dynamic regulation of both histone (e.g., H3K18-la, H3K14-la) and non-histone (e.g., ACSF2, Fis1) proteins. Lactylation is context-dependent, particularly at the critical residue H3K18-la, whose function varies with disease. In the ccRCC tumor microenvironment, H3K18-la mainly promotes transcription of oncogenic genes such as PDGFRβ, thereby driving tumor progression and enabling immune evasion. By contrast, in injury settings such as AKI and CKD, H3K18-la amplifies tissue inflammation, fibrosis, and cellular damage by activating inflammation- and fibrosis-related pathways, including NF-κB, TGF-β/Smad, and EMT. This functional divergence helps explain the distinct pathological roles of lactylation in renal cell carcinoma versus other kidney diseases. The differing mechanisms are summarized in Figure 2.

Notably, while most studies emphasize the disease-promoting effects of lactylation, the discovery of the HSPA12A/c-Myc axis reveals a protective role in IRI-AKI by promoting tubular epithelial cell proliferation, highlighting both the complexity and challenge in understanding its pathophysiological context. However, several critical challenges remain. The specific regulatory mechanisms of lactylation, particularly its differential expression patterns and functions in various renal cell types (e.g., podocytes, tubular epithelial cells), require further investigation. Additionally, the crosstalk between lactylation and other PTMs (e.g., acetylation, phosphorylation) and their collective impact on kidney disease progression demands systematic exploration. Lactylation does not function in isolation. p300 serves as a shared “writer” for both lactylation and acetylation, while HDAC1–3 and SIRT1–3 act as dual “erasers” for both modifications. In non-renal diseases such as cancer, direct competition between lactylation and acetylation, as well as other short-chain acylations, at shared lysine residues has been well documented. However, in the context of renal fibrosis, AKI, or DKD, sufficient functional validation is still lacking. Rigorously distinguishing their actual contributions to lactylation, acetylation, and other short-chain acylations in disease animal models is essential to avoid misattributing the combined effects of multiple modifications to lactylation alone as a unitary therapeutic effect. At the non-histone level, lactylation at K91 of TFEB has been shown to inhibit its ubiquitination and proteasomal degradation by blocking binding to the E3 ubiquitin ligase WWP2, revealing a novel paradigm in which lactylation directly interferes with ubiquitination (Huang et al., 2024). The applicability of this mechanism in kidney diseases also warrants further exploration. Future studies should combine site-directed mutagenesis with multi-omics approaches to systematically map the multilayered PTM crosstalk networks, thereby providing a foundation for the development of precision intervention strategies.

Translating targeted lactylation from basic research into clinical practice is an urgent and unresolved challenge. Lactylation links metabolic disturbances with epigenetic remodeling in DKD and constitutes a central driver of disease initiation and progression. Thus, targeting the “lactate-lactylation axis” represents a promising therapeutic strategy. Many traditional Chinese medicines—both compound formulas and isolated monomers—have demonstrated benefits for glucose metabolism, inflammation, oxidative stress, and fibrosis (Chen X. et al., 2024). These natural products may lower lactate production by inhibiting glycolytic enzymes such as PKM2, LDHA, and PFKFB3, or they may modulate the activity of lactylation “writers” and “erasers,” thereby precisely intervening at this metabolic–epigenetic nexus. This perspective offers a new molecular framework for understanding how traditional Chinese medicine protects the kidney in DKD by integrating metabolic reprogramming and epigenetic modification. In advancing clinical translation, it is essential to maintain a clear appraisal of potential risks. Because glycolysis is a central metabolic pathway present throughout the body, systemic inhibition of glycolytic enzymes (for example, HK2 or PKM2 inhibitors) could produce severe off-target metabolic toxicity and impair the function of high–energy–demand organs such as the heart and brain. A fundamental question thus arises: can systemic toxicity be circumvented through optimized administration routes or judicious target selection? The answer may lie in the rational selection of therapeutic targets at an appropriate hierarchical level. Future targeted interventions should focus on specifically inhibiting novel lactyltransferases (such as utilizing β-alanine to competitively inhibit AARS1/2) or employing kidney-targeted nanocarriers to modulate lactylation locally, thereby avoiding systemic metabolic disruption. avoiding systemic metabolic disruption while circumventing the metabolic toxicity associated with global glycolytic inhibition. This strategic shift from “pathway inhibitors” to “modification enzyme inhibitors” constitutes the core direction of precision intervention. Moreover, the functional role of lactylation is not unambiguously “beneficial” or “detrimental.” In the context of post-myocardial infarction repair, lactylation promotes the activation of reparative genes, while in the IRI-AKI, the HSPA12A/c-Myc axis has been shown to exert a protective effect by promoting tubular epithelial cell proliferation (Wang N. et al., 2022). Collectively, these findings provide a rationale for designing therapeutic interventions that are contingent on time windows and tissue specificity. Therefore, future therapeutic strategies may need to focus on addressing the spatiotemporal specificity of lactylation, achieving appropriate regulation at the right time and in the right tissue, rather than simply inhibiting or activating it in a global manner.

Detection technologies have improved relative to the past, but important limitations remain. Pan-Kla antibodies and several site-specific antibodies (for example, H3K18-la) have been developed, yet their utility is restricted, and lactylation itself is a highly dynamic, metabolism-linked modification. Mass spectrometry and antibody-based methods only capture a static snapshot at a single time point. Emerging fluorescent probes and artificial intelligence–based prediction tools provide promising new directions, but each has drawbacks: fluorescent probes still require rigorous validation of specificity, sensitivity, and scalability, while AI approaches serve mainly as preliminary screening tools that demand experimental confirmation and currently suffer from limited prediction accuracy. Future research should focus on developing novel detection technologies to precisely track the dynamic changes in lactylation across different renal regions and disease stages. Investigating its precise regulatory mechanisms in kidney development, homeostasis, and disease pathogenesis, exploring its potential as non-invasive diagnostic biomarkers, and designing specific intervention strategies targeting lactylation pathways are essential steps. With these advancements, lactylation modification may emerge as a potential strategy for the precision diagnosis and treatment of kidney diseases, offering additional therapeutic avenues.

## Glossary

PTM: post-translational modification
DKD: diabetic kidney disease
ccRCC: clear cell renal cell carcinoma
AKI: acute kidney injury
CKD: chronic kidney disease
MCTs: monocarboxylate transporters
TCA cycle: the tricarboxylic acid
LDH: lactate dehydrogenase
PDH: pyruvate dehydrogenase
Bcl2l11: the pro-apoptotic gene Bcl2-like 11
SIRT1: Sirtuin 1
FOXO4: Forkhead box O4
STAT3: Signal transducer and activator of transcription 3
EMT: epithelial-mesenchymal transition
AMWAP: activated microglia/macrophage WAP domain protein
Kcr: lysine crotonylation
Kac: lysine acetylation
ACSS2: acyl-CoA synthetase short-chain family member 2
KAT2A: lysine acetyltransferase 2A
H3K18-la: histone H3 lysine 18 lactylation
FGFR4: fibroblast growth factor receptor 4
PDGFRβ: the platelet-derived growth factor receptor-β
GAL3: galectin-3
PKM2: pyruvate kinase M2
FKBP10: FK506-binding protein 10
LDHA: lactate dehydrogenase A
PFKFB3: 6- phosphofructo-2-kinase/fructose-2,6-bisphosphatase 3
(CycIm) ions: cyclic immonium
Kla: lysine lactylation
ALDH2: aldehyde dehydrogenase 2
HK-2: human renal proximal tubular cells
ACSF2: acyl-CoA synthetase short-chain family member 2
RTEC: renal tubular epithelial cell
mtROS: mitochondrial reactive oxygen species
IGFBP5: insulin-like growth factor binding protein 5
KLF5: the Krüppel-like factor 5
GLIS1: GLIS family zinc finger 1
KAT5: lysine acetyltransferase 5
KDIGO: the Kidney Disease: Improving Global Outcomes
SCr: serum creatinine
ICUs: intensive care units
SIRT3: Sirtuin 3
PDHA1: pyruvate dehydrogenase E1 component subunit alpha
Y10: tyrosine 10
ALDH2: aldehyde dehydrogenase 2
Kla: pan-histone lactylation
HMGB1: high mobility group box 1
cGAS: the cyclic GMP-AMP synthase
STING: stimulator of interferon genes
mtDNA: mitochondrial DNA
DAMPs: damage-associated molecular patterns
NET: neutrophil extracellular trap
IRAK1: interleukin-1 receptor-associated kinase 1
IRI: Ischemia-reperfusion injury
HK2: Hexokinase 2
HSPA12A: heat shock protein A12A
HIF1α: the hypoxia-inducible factor 1α
NCCN: National Comprehensive Cancer Network
VHL: the von Hippel-Lindau
TME: tumor microenvironment
HIBCH: 3- hydroxyisobutyryl-CoA hydrolase
BRD: bromodomain-containing protein
NAT10: N-acetyltransferase 10
KLF6: Krüppel-like factor 6
ENTPD5: ectonucleoside triphosphate diphosphohydrolase 5
ER: endoplasmic reticulum
IgAN: IgA nephropathy
IgA1: immunoglobulin A1
FADD: Fas-associated protein with death domain
SMAD: mothers against decapentaplegic homolog
PAD3: peptidylarginine deiminase 3
p38 MAPK: p38 mitogen-activated protein kinase
EGFR: epidermal growth factor receptor
AMPK: AMP-activated protein kinase
PFKFB2: 6-phosphofructo-2-kinase/fructose-2,6-bisphosphatase 2
IL-1β: interleukin-1 beta
AARS1: alanyl-tRNA synthetase 1
ELOVL5: ELOVL fatty acid elongase 5
ACSL4: acyl-CoA synthetase long-chain 4
PFKM: phosphofructokinase muscle type
TMAO: trimethylamine N-oxide
CS: Citrate Synthase
NLRP3: NLR family pyrin domain containing 3
HDAC1: histone deacetylase 1
Kl-la: Lysine L-lactylation
Kce: N-ε-(carboxyethyl)-lysine
Kd-la: D-lactyl-lysine
cGAS: the cyclic GMP-AMP synthase
GTPSCS: GTP-specific succinyl-CoA synthetase

## References

[B1] Adeva-Andany M. López-Ojén M. Funcasta-Calderón R. Ameneiros-Rodríguez E. Donapetry-García C. Vila-Altesor M. (2014). Comprehensive review on lactate metabolism in human health. Mitochondrion 17, 76–100. 10.1016/j.mito.2014.05.007 24929216

[B2] Alicic R. Z. Rooney M. T. Tuttle K. R. (2017). Diabetic kidney disease: challenges, progress, and possibilities. Clin. J. Am Soc Nephrol 12 (12), 2032–2045. 10.2215/CJN.11491116 28522654 PMC5718284

[B3] An S. Yao Y. Hu H. Wu J. Li J. Li L. (2023). Pdha1 hyperacetylation-mediated lactate overproduction promotes sepsis-induced acute kidney injury via fis1 lactylation. Cell Death Dis 14 (7), 457. 10.1038/s41419-023-05952-4 37479690 PMC10362039

[B4] Anraku T. Murata M. Kuroki H. Kazama A. Shirono Y. Tasaki M. (2024). Selective hdac6 inhibition has the potential for anti-cancer effect in renal cell carcinoma. J. Pers Med 14 (7), 704. 10.3390/jpm14070704 39063958 PMC11278056

[B5] Bellomo R. (2002). Bench-to-bedside review: lactate and the kidney. Crit. Care 6 (4), 322–326. 10.1186/cc1518 12225607 PMC137458

[B6] Benjamin D. Robay D. Hindupur S. K. Pohlmann J. Colombi M. El-Shemerly M. Y. (2018). Dual inhibition of the lactate transporters mct1 and mct4 is synthetic lethal with metformin due to nad+ depletion in cancer cells. Cell Rep 25 (11), 3047–3058.e3044. 10.1016/j.celrep.2018.11.043 30540938 PMC6302548

[B7] Bhargava P. Schnellmann R. G. (2017). Mitochondrial energetics in the kidney. Nat. Rev. Nephrol. 13 (10), 629–646. 10.1038/nrneph.2017.107 28804120 PMC5965678

[B8] Brooks G. A. (2009). Cell-cell and intracellular lactate shuttles. J. Physiol. 587 (23), 5591–5600. 10.1113/jphysiol.2009.178350 19805739 PMC2805372

[B9] Brooks G. A. (2018). The science and translation of lactate shuttle theory. Cell Metab. 27 (4), 757–785. 10.1016/j.cmet.2018.03.008 29617642

[B10] Chang-Panesso M. Kadyrov F. F. Lalli M. Wu H. Ikeda S. Kefaloyianni E. (2019). Foxm1 drives proximal tubule proliferation during repair from acute ischemic kidney injury. J. Clin Invest 129 (12), 5501–5517. 10.1172/JCI125519 31710314 PMC6877314

[B11] Chen Y. Zhu Y. Sheng Y. Xiao J. Xiao Y. Cheng N. (2019). Sirt1 downregulated fgb expression to inhibit rcc tumorigenesis by destabilizing stat3. Exp. Cell Res 382 (2), 111466. 10.1016/j.yexcr.2019.06.011 31201813

[B12] Chen J. Feng Q. Qiao Y. Pan S. Liang L. Liu Y. (2024a). Acsf2 and lysine lactylation contribute to renal tubule injury in diabetes. Diabetologia 67 (7), 1429–1443. 10.1007/s00125-024-06156-x 38676722

[B13] Chen X. Li X. Cao B. Chen X. Zhang K. Han F. (2024b). Mechanisms and efficacy of traditional Chinese herb monomers in diabetic kidney disease. Int. Urol. Nephrol. 56 (2), 571–582. 10.1007/s11255-023-03703-0 37552392

[B14] Chen J. He J. Wang X. Bai L. Yang X. Chen J. (2025). Glis1 inhibits rtec cellular senescence and renal fibrosis by downregulating histone lactylation in dkd. Life Sci 361, 123293. 10.1016/j.lfs.2024.123293 39643036

[B15] Chen Y. Wang W. Cheng M. Wei L. Sang Y. Gao Y. (2026). The glycolytic enzyme PFKM promotes renal fibrosis by activating the NF-κB pathway via lactate-mediated H3K18 lactylation. Cell Mol. Life Sci. 83 (1), 121. 10.1007/s00018-026-06118-z 41711888 PMC12932785

[B16] Chiba T. Peasley K. D. Cargill K. R. Maringer K. V. Bharathi S. S. Mukherjee E. (2019). Sirtuin 5 regulates proximal tubule fatty acid oxidation to protect against aki. J. Am Soc Nephrol 30 (12), 2384–2398. 10.1681/ASN.2019020163 31575700 PMC6900790

[B17] Corridon P. R. (2023). Enhancing the expression of a key mitochondrial enzyme at the inception of ischemia-reperfusion injury can boost recovery and halt the progression of acute kidney injury. Front. Physiol 14, 1024238. 10.3389/fphys.2023.1024238 36846323 PMC9945300

[B18] Coughlan A. Y. Testa G. (2021). Exploiting epigenetic dependencies in ovarian cancer therapy. Int. J. Cancer 149 (10), 1732–1743. 10.1002/ijc.33727 34213777 PMC9292863

[B19] Darshi M. Kugathasan L. Maity S. Sridhar V. S. Fernandez R. Limonte C. P. (2024). Glycolytic lactate in diabetic kidney disease. JCI Insight 9 (11), e168825. 10.1172/jci.insight.168825 38855868 PMC11382878

[B20] Drabkin M. Yogev Y. Zeller L. Zarivach R. Zalk R. Halperin D. (2019). Hyperuricemia and gout caused by missense mutation in d-lactate dehydrogenase. J. Clin. Invest 129 (12), 5163–5168. 10.1172/JCI129057 31638601 PMC6877321

[B21] Du Y. W. Li X. K. Wang T. T. Zhou L. Li H. R. Feng L. (2023). Cyanidin-3-glucoside inhibits ferroptosis in renal tubular cells after ischemia/reperfusion injury via the ampk pathway. Mol. Med 29 (1), 42. 10.1186/s10020-023-00642-5 37013504 PMC10069074

[B22] Ferenbach D. A. Bonventre J. V. (2015). Mechanisms of maladaptive repair after aki leading to accelerated kidney ageing and ckd. Nat. Rev Nephrol 11 (5), 264–276. 10.1038/nrneph.2015.3 25643664 PMC4412815

[B23] Ferguson B. S. Rogatzki M. J. Goodwin M. L. Kane D. A. Rightmire Z. Gladden L. B. (2018). Lactate metabolism: historical context, prior misinterpretations, and current understanding. Eur. J. Appl Physiol 118 (4), 691–728. 10.1007/s00421-017-3795-6 29322250

[B24] Fukushi A. Kim H. D. Chang Y. C. Kim C. H. (2022). Revisited metabolic control and reprogramming cancers by means of the warburg effect in tumor cells. Int. J. Mol Sci 23 (17), 10037. 10.3390/ijms231710037 36077431 PMC9456516

[B25] Gaffney D. O. Jennings E. Q. Anderson C. C. Marentette J. O. Shi T. Schou Oxvig A. M. (2020). Non-enzymatic lysine lactoylation of glycolytic enzymes. Cell Chem Biol 27 (2), 206–213.e206. 10.1016/j.chembiol.2019.11.005 31767537 PMC7395678

[B26] Guzzi F. Cirillo L. Roperto R. M. Romagnani P. Lazzeri E. (2019). Molecular mechanisms of the acute kidney injury to chronic kidney disease transition: an updated view. Int. J. Mol Sci. 20 (19), 4941. 10.3390/ijms20194941 31590461 PMC6801733

[B27] Hagihara H. Shoji H. Otabi H. Toyoda A. Katoh K. Namihira M. (2021). Protein lactylation induced by neural excitation. Cell Rep 37 (2), 109820. 10.1016/j.celrep.2021.109820 34644564

[B28] Hasegawa K. Wakino S. Simic P. Sakamaki Y. Minakuchi H. Fujimura K. (2013). Renal tubular sirt1 attenuates diabetic albuminuria by epigenetically suppressing claudin-1 overexpression in podocytes. Nat. Med 19 (11), 1496–1504. 10.1038/nm.3363 24141423 PMC4041199

[B29] Heilman R. L. Smith M. L. Kurian S. M. Huskey J. Batra R. K. Chakkera H. A. (2015). Transplanting kidneys from deceased donors with severe acute kidney injury. Am. J. Transpl. 15 (8), 2143–2151. 10.1111/ajt.13260 25808278

[B30] Hong Q. Zhang L. Das B. Li Z. Liu B. Cai G. (2018). Increased podocyte sirtuin-1 function attenuates diabetic kidney injury. Kidney Int. 93 (6), 1330–1343. 10.1016/j.kint.2017.12.008 29477240 PMC5967974

[B31] Hong J. Xu H. Yu L. Yu Z. Chen X. Meng Z. (2026). AARS1-mediated lactylation of H3K18 and STAT1 promotes ferroptosis in diabetic nephropathy. Cell Death Differ. 33 (3), 589–604. 10.1038/s41418-025-01587-4 40987895 PMC13036035

[B32] Hu K. Yu Z. Yuan Y. Yi J. Li J. Zhu M. (2025). Lactate/AARS1/H3K18la/LDHA positive feedback loop triggers ferroptosis, which participates in diabetic nephropathy via the modulation of ACSL4 transcription. Acta Biochim. Biophys. Sin. (Shanghai). 10.3724/abbs.2025200 41169201

[B33] Hu X. Chen W. Yang M. Li M. Li X. Ouyang S. (2025). Igfbp5 promotes endomt and renal fibrosis through h3k18 lactylation in diabetic nephropathy. Cell Mol Life Sci 82 (1), 215. 10.1007/s00018-025-05718-5 40423799 PMC12116956

[B34] Huang Y. Luo G. Peng K. Song Y. Wang Y. Zhang H. (2024). Lactylation stabilizes TFEB to elevate autophagy and lysosomal activity. J. Cell Biol. 223 (11), e202308099. 10.1083/jcb.202308099 39196068 PMC11354204

[B35] Huang Z. Liao Y. Zheng Y. Ye S. Zhang Q. Yu X. (2025). Zinc deficiency causes glomerulosclerosis and renal interstitial fibrosis through oxidative stress and increased lactate metabolism in rats. Biol. Trace Elem. Res. 203 (4), 2084–2098. 10.1007/s12011-024-04306-1 39028478 PMC11919932

[B36] Irizarry-Caro R. A. McDaniel M. M. Overcast G. R. Jain V. G. Troutman T. D. Pasare C. (2020). Tlr signaling adapter bcap regulates inflammatory to reparatory macrophage transition by promoting histone lactylation. Proc. Natl Acad Sci U. S. A. 117 (48), 30628–30638. 10.1073/pnas.2009778117 33199625 PMC7720107

[B37] Ji L. Chen Y. Wang H. Zhang W. He L. Wu J. (2019). Overexpression of sirt6 promotes m2 macrophage transformation, alleviating renal injury in diabetic nephropathy. Int. J. Oncol 55 (1), 103–115. 10.3892/ijo.2019.4800 31115579 PMC6561622

[B38] Jiang N. Niu G. Pan Y. H. Pan W. Zhang M. F. Zhang C. Z. (2020). Cbx4 transcriptionally suppresses klf6 via interaction with hdac1 to exert oncogenic activities in clear cell renal cell carcinoma. Ebiomedicine 53, 102692. 10.1016/j.ebiom.2020.102692 32113161 PMC7044754

[B39] Ju J. Zhang H. Lin M. Yan Z. An L. Cao Z. (2024). The alanyl-tRNA synthetase AARS1 moonlights as a lactyltransferase to promote YAP signaling in gastric cancer. J. Clin. Invest 134 (10), e174587. 10.1172/JCI174587 38512451 PMC11093599

[B40] Jung Y. J. Lee A. S. Nguyen-Thanh T. Kim D. Kang K. P. Lee S. (2015). Sirt2 regulates lps-induced renal tubular cxcl2 and ccl2 expression. J. Am Soc Nephrol 26 (7), 1549–1560. 10.1681/ASN.2014030226 25349202 PMC4483578

[B41] KDIGO (2024). Clinical practice guideline for the evaluation and management of chronic kidney disease. Kidney Int. 105 (4), S117–s314. 10.1016/j.kint.2023.10.018 38490803

[B42] Khan M. M. Paez H. G. Pitzer C. R. Alway S. E. (2023). The therapeutic potential of mitochondria transplantation therapy in neurodegenerative and neurovascular disorders. Curr. Neuropharmacol. 21 (5), 1100–1116. 10.2174/1570159X05666220908100545 36089791 PMC10286589

[B43] Kim S. G. Lee J. Yun D. Kang M. W. Kim Y. C. Kim D. K. (2023). Hyperlactatemia is a predictor of mortality in patients undergoing continuous renal replacement therapy for acute kidney injury. BMC Nephrol. 24 (1), 11. 10.1186/s12882-023-03063-y 36641421 PMC9840420

[B44] Kim H. Park S. Y. Lee S. Y. Kwon J. H. Byun S. Ko B. (2025). Loss of p300 in proximal tubular cells reduces renal fibrosis and endothelial-mesenchymal transition. Embo Mol Med 17 (7), 1575–1598. 10.1038/s44321-025-00243-1 40588562 PMC12254316

[B45] Kobayashi M. Narumi K. Furugen A. Iseki K. (2021). Transport function, regulation, and biology of human monocarboxylate transporter 1 (hmct1) and 4 (hmct4). Pharmacol. Ther 226, 107862. 10.1016/j.pharmthera.2021.107862 33894276

[B46] Lafrance J. P. Miller D. R. (2010). Acute kidney injury associates with increased long-term mortality. J. Am Soc Nephrol 21 (2), 345–352. 10.1681/ASN.2009060636 20019168 PMC2834549

[B47] Lee M. Harley G. Katerelos M. Gleich K. Sullivan M. A. Laskowski A. (2020). Mutation of regulatory phosphorylation sites in pfkfb2 worsens renal fibrosis. Sci. Rep 10 (1), 14531. 10.1038/s41598-020-71475-z 32884050 PMC7471692

[B48] Legouis D. Faivre A. Cippà P. E. de Seigneux S. (2022). Renal gluconeogenesis: an underestimated role of the kidney in systemic glucose metabolism. Nephrol. Dial Transplant 37 (8), 1417–1425. 10.1093/ndt/gfaa302 33247734

[B49] Li X. Zhuang S. (2014). Recent advances in renal interstitial fibrosis and tubular atrophy after kidney transplantation. Fibrogenes. Tissue Repair 7, 15. 10.1186/1755-1536-7-15 25285155 PMC4185272

[B50] Li Z. Xu K. Zhang N. Amador G. Wang Y. Zhao S. (2018). Overexpressed sirt6 attenuates cisplatin-induced acute kidney injury by inhibiting erk1/2 signaling. Kidney Int 93 (4), 881–892. 10.1016/j.kint.2017.10.021 29373150

[B51] Li Z. Lu S. Li X. (2021). The role of metabolic reprogramming in tubular epithelial cells during the progression of acute kidney injury. Cell Mol Life Sci 78 (15), 5731–5741. 10.1007/s00018-021-03892-w 34185125 PMC11073237

[B52] Li X. Yang Y. Zhang B. Lin X. Fu X. An Y. (2022). Lactate metabolism in human health and disease. Signal Transduct Target Ther 7 (1), 305. 10.1038/s41392-022-01151-3 36050306 PMC9434547

[B53] Li J. Yu C. Shen F. Cui B. Liu N. Zhuang S. (2022). Class iia histone deacetylase inhibition ameliorates acute kidney injury by suppressing renal tubular cell apoptosis and enhancing autophagy and proliferation. Front. Pharmacol 13, 946192. 10.3389/fphar.2022.946192 35935816 PMC9354984

[B54] Li L. Feng Y. Zhang J. Zhang Q. Ren J. Sun C. (2022). Microtubule associated protein 4 phosphorylation-induced epithelial-to-mesenchymal transition of podocyte leads to proteinuria in diabetic nephropathy. Cell Commun Signal 20 (1), 115. 10.1186/s12964-022-00883-7 35902952 PMC9331595

[B55] Li J. Huang X. He K. Wu S. (2023). The kidney antifibrotic effects of 5,7,3',4',5'-pentamethoxyflavone from bauhinia championii in streptozotocin-induced diabetic rats: *in vivo* and *in vitro* experiments. Pharm. Biol 61 (1), 938–948. 10.1080/13880209.2023.2222773 37345554 PMC10288925

[B56] Li L. Xiang T. Guo J. Guo F. Wu Y. Feng H. (2024). Inhibition of acss2-mediated histone crotonylation alleviates kidney fibrosis via il-1β-dependent macrophage activation and tubular cell senescence. Nat. Commun 15 (1), 3200. 10.1038/s41467-024-47315-3 38615014 PMC11016098

[B57] Li H. Liu C. Li R. Zhou L. Yang Q. (2024). AARS1 and AARS2 sense L-lactate to regulate cGAS as global lysine lactyltransferases. Nature 634 (8036), 1229–1237. 10.1038/s41586-024-07992-y 39322678

[B58] Li Y. Min X. Zhang X. Cao X. Kong Q. Mao Q. (2024). Hspa12a promotes c-myc lactylation-mediated proliferation of tubular epithelial cells to facilitate renal functional recovery from kidney ischemia/reperfusion injury. Cell Mol Life Sci. 81 (1), 404. 10.1007/s00018-024-05427-5 39277835 PMC11402889

[B59] Li J. Shi X. Xu J. Wang K. Hou F. Luan X. (2025). Aldehyde dehydrogenase 2 lactylation aggravates mitochondrial dysfunction by disrupting phb2 mediated mitophagy in acute kidney injury. Adv. Sci (weinh) 12 (8), e2411943. 10.1002/advs.202411943 39737891 PMC11848585

[B60] Lin Y. Cai F. Wang X. Yang Y. Ren Y. Yao C. (2023a). Fadd phosphorylation contributes to development of renal fibrosis by accelerating epithelial-mesenchymal transition. Cell Cycle 22 (5), 580–595. 10.1080/15384101.2022.2136463 36281535 PMC9928456

[B61] Lin Y. Wu P. Guo L. Feng Q. Wang L. Lin X. (2023b). Prevalence of diabetic kidney disease with different subtypes in hospitalized patients with diabetes and correlation between egfr and lncrna xist expression in pbmcs. Diabetes Ther 14 (9), 1549–1561. 10.1007/s13300-023-01439-9 37422842 PMC10363095

[B62] Liu J. Zhou F. Tang Y. Li L. Li L. (2024). Progress in lactate metabolism and its regulation via small molecule drugs. Molecules 29 (23), 5656. 10.3390/molecules29235656 39683818 PMC11643809

[B63] Liu R. Zou Z. Chen L. Feng Y. Ye J. Deng Y. (2024). Fkbp10 promotes clear cell renal cell carcinoma progression and regulates sensitivity to the hif2α blockade by facilitating ldha phosphorylation. Cell Death Dis 15 (1), 64. 10.1038/s41419-024-06450-x 38233415 PMC10794466

[B64] Liu R. Ren X. Park Y. E. Feng H. Sheng X. Song X. (2025). Nuclear GTPSCS functions as a lactyl-CoA synthetase to promote histone lactylation and gliomagenesis. Cell Metab. 37 (2), 377–394.e379. 10.1016/j.cmet.2024.11.005 39642882 PMC11798710

[B65] Liu Y. Yan Z. Liu C. Yang R. Zheng Q. Jian J. (2025). Integrated RNA sequencing analysis and machine learning identifies a metabolism-related prognostic signature in clear cell renal cell carcinoma. Sci. Rep. 15 (1), 1691. 10.1038/s41598-025-85618-7 39799252 PMC11724983

[B66] Luo X. Deng L. Lamsal L. P. Xu W. Xiang C. Cheng L. (2015). Amp-activated protein kinase alleviates extracellular matrix accumulation in high glucose-induced renal fibroblasts through mtor signaling pathway. Cell Physiol. Biochem 35 (1), 191–200. 10.1159/000369687 25591762

[B67] Martinez-Moreno J. M. Fontecha-Barriuso M. Martín-Sánchez D. Sánchez-Niño M. D. Ruiz-Ortega M. Sanz A. B. (2020). The contribution of histone crotonylation to tissue health and disease: focus on kidney health. Front. Pharmacol 11, 393. 10.3389/fphar.2020.00393 32308622 PMC7145939

[B68] Matsushita K. Ballew S. H. Wang A. Y. Kalyesubula R. Schaeffner E. Agarwal R. (2022). Epidemiology and risk of cardiovascular disease in populations with chronic kidney disease. Nat. Rev Nephrol 18 (11), 696–707. 10.1038/s41581-022-00616-6 36104509

[B69] Miguel V. Kramann R. (2023). Metabolic reprogramming heterogeneity in chronic kidney disease. FEBS Open Bio 13 (7), 1154–1163. 10.1002/2211-5463.13568 36723270 PMC10315765

[B70] Millán-Zambrano G. Burton A. Bannister A. J. Schneider R. (2022). Histone post-translational modifications - cause and consequence of genome function. Nat. Rev Genet 23 (9), 563–580. 10.1038/s41576-022-00468-7 35338361

[B71] Moradi H. Wang P. H. (2013). Renoprotective mechanisms of ischemic postconditioning in ischemia-reperfusion injury: improved mitochondrial function and integrity. Nephrol. Dial Transplant 28 (11), 2667–2669. 10.1093/ndt/gft313 24169608 PMC3811058

[B72] Moreno-Yruela C. Zhang D. Wei W. Bæk M. Liu W. Gao J. (2022). Class I histone deacetylases (HDAC1-3) are histone lysine delactylases. Sci. Adv. 8 (3), eabi6696. 10.1126/sciadv.abi6696 35044827 PMC8769552

[B73] Motzer R. J. Jonasch E. Agarwal N. Alva A. Baine M. Beckermann K. (2022). Kidney cancer, version 3.2022, nccn clinical practice guidelines in oncology. J. Natl Compr Canc Netw 20 (1), 71–90. 10.6004/jnccn.2022.0001 34991070 PMC10191161

[B74] Panizo S. Martínez-Arias L. Alonso-Montes C. Cannata P. Martín-Carro B. Fernández-Martín J. L. (2021). Fibrosis in chronic kidney disease: pathogenesis and consequences. Int. J. Mol. Sci. 22 (1), 408. 10.3390/ijms22010408 33401711 PMC7795409

[B75] Patel J. H. Loboda A. P. Showe M. K. Showe L. C. McMahon S. B. (2004). Analysis of genomic targets reveals complex functions of myc. Nat. Rev Cancer 4 (7), 562–568. 10.1038/nrc1393 15229481

[B76] Peerapornratana S. Manrique-Caballero C. L. Gómez H. Kellum J. A. (2019). Acute kidney injury from sepsis: current concepts, epidemiology, pathophysiology, prevention and treatment. Kidney Int 96 (5), 1083–1099. 10.1016/j.kint.2019.05.026 31443997 PMC6920048

[B77] Perazella M. A. Rosner M. H. (2022). Drug-induced acute kidney injury. Clin. J. Am Soc Nephrol 17 (8), 1220–1233. 10.2215/cjn.11290821 35273009 PMC9435983

[B78] Qi B. Chen Y. Chai S. Lu X. Kang L. (2025). O-linked β-n-acetylglucosamine (o-glcnac) modification: emerging pathogenesis and a therapeutic target of diabetic nephropathy. Diabet. Med 42 (2), e15436. 10.1111/dme.15436 39279604 PMC11733667

[B79] Qiao J. Tan Y. Liu H. Yang B. Zhang Q. Liu Q. (2024). Histone h3k18 and ezrin lactylation promote renal dysfunction in sepsis-associated acute kidney injury. Adv. Sci (weinh) 11 (28), e2307216. 10.1002/advs.202307216 38767134 PMC11267308

[B80] Ranganathan P. Hamad R. Mohamed R. Jayakumar C. Muthusamy T. Ramesh G. (2016). Histone deacetylase–mediated silencing of amwap expression contributes to cisplatin nephrotoxicity. Kidney Int 89 (2), 317–326. 10.1038/ki.2015.326 26509586 PMC4848209

[B81] Sanchez D. J. Simon M. C. (2018). Genetic and metabolic hallmarks of clear cell renal cell carcinoma. Biochim. Biophys. Acta Rev. Cancer 1870 (1), 23–31. 10.1016/j.bbcan.2018.06.003 29959988 PMC6561085

[B82] Scheijen J. L. Hanssen N. M. van de Waarenburg M. P. Jonkers D. M. Stehouwer C. D. Schalkwijk C. G. (2012). L(+) and D(-) lactate are increased in plasma and urine samples of type 2 diabetes as measured by a simultaneous quantification of L(+) and D(-) lactate by reversed-phase liquid chromatography tandem mass spectrometry. Exp. Diabetes Res. 2012, 234812. 10.1155/2012/234812 22474418 PMC3310144

[B83] Shen H. Holliday M. Sheikh-Hamad D. Li Q. Tong Q. Hamad C. D. (2021). Sirtuin-3 mediates sex differences in kidney ischemia-reperfusion injury. Transl. Res 235, 15–31. 10.1016/j.trsl.2021.03.015 33789208

[B84] Sun H. J. Xiong S. P. Cao X. Cao L. Zhu M. Y. Wu Z. Y. (2021). Polysulfide-mediated sulfhydration of sirt1 prevents diabetic nephropathy by suppressing phosphorylation and acetylation of p65 nf-κb and stat3. Redox Biol. 38, 101813. 10.1016/j.redox.2020.101813 33279869 PMC7718489

[B85] Sun M. Li J. Mao L. Wu J. Deng Z. He M. (2021). P53 deacetylation alleviates sepsis-induced acute kidney injury by promoting autophagy. Front. Immunol 12, 685523. 10.3389/fimmu.2021.685523 34335587 PMC8318785

[B86] Sun Z. Wang Y. Zheng C. Xiao L. Zang Y. Fang L. (2025). Nat10 promotes the progression of clear cell renal cell carcinoma by regulating ac4c acetylation of nfe2l3 and activating akt/gsk3β signaling pathway. Cell Death Dis 16 (1), 235. 10.1038/s41419-025-07528-w 40169553 PMC11962090

[B87] Tang Y. Li Y. Yang X. Lu T. Wang X. Li Z. (2026). Intestinal metabolite TMAO promotes CKD progression by stimulating macrophage M2 polarization through histone H4 lysine 12 lactylation. Cell Death Differ. 33 (2), 314–326. 10.1038/s41418-025-01554-z 40830246 PMC12881611

[B88] Tawengi M. Al-Dali Y. Tawengi A. Benter I. F. Akhtar S. (2024). Targeting the epidermal growth factor receptor (egfr/erbb) for the potential treatment of renal pathologies. Front. Pharmacol 15, 1394997. 10.3389/fphar.2024.1394997 39234105 PMC11373609

[B89] Trujillo M. N. Jennings E. Q. Hoffman E. A. Zhang H. Phoebe A. M. Mastin G. E. (2024). Lactoylglutathione promotes inflammatory signaling in macrophages through histone lactoylation. Mol. Metab. 81, 101888. 10.1016/j.molmet.2024.101888 38307385 PMC10869261

[B90] Varner E. L. Trefely S. Bartee D. von Krusenstiern E. Izzo L. Bekeova C. (2020). Quantification of lactoyl-CoA (lactyl-CoA) by liquid chromatography mass spectrometry in mammalian cells and tissues. Open Biol. 10 (9), 200187. 10.1098/rsob.200187 32961073 PMC7536085

[B91] Venkatachalam M. A. Weinberg J. M. Kriz W. Bidani A. K. (2015). Failed tubule recovery, aki-ckd transition, and kidney disease progression. J. Am Soc Nephrol 26 (8), 1765–1776. 10.1681/ASN.2015010006 25810494 PMC4520181

[B92] Wang X. Fan W. Li N. Ma Y. Yao M. Wang G. (2023). Yy1 lactylation in microglia promotes angiogenesis through transcription activation-mediated upregulation of fgf2. Genome Biol 24 (1), 87. 10.1186/s13059-023-02931-y 37085894 PMC10120156

[B93] Wan N. Wang N. Yu S. Zhang H. Tang S. Wang D. (2022). Cyclic immonium ion of lactyllysine reveals widespread lactylation in the human proteome. Nat. Methods 19 (7), 854–864. 10.1038/s41592-022-01523-1 35761067

[B94] Wang X. Liu J. Zhen J. Zhang C. Wan Q. Liu G. (2014). Histone deacetylase 4 selectively contributes to podocyte injury in diabetic nephropathy. Kidney Int 86 (4), 712–725. 10.1038/ki.2014.111 24717296

[B95] Wang L. Jiao L. Pang S. Yan P. Wang X. Qiu T. (2021). The development of design and manufacture techniques for bioresorbable coronary artery stents. Micromachines (Basel) 12 (8), 990. 10.3390/mi12080990 34442612 PMC8398368

[B96] Wang, J J. Yang P. Yu T. Gao M. Liu D. Zhang J. (2022). Lactylation of pkm2 suppresses inflammatory metabolic adaptation in pro-inflammatory macrophages. Int. J. Biol Sci 18 (16), 6210–6225. 10.7150/ijbs.75434 36439872 PMC9682528

[B97] Wang, S S. Chen Y. Han S. Liu Y. Gao J. Huang Y. (2022). Selenium nanoparticles alleviate ischemia reperfusion injury-induced acute kidney injury by modulating gpx-1/nlrp3/caspase-1 pathway. Theranostics 12 (8), 3882–3895. 10.7150/thno.70830 35664065 PMC9131270

[B98] Wang, N N. Wang W. Wang X. Mang G. Chen J. Yan X. (2022). Histone lactylation boosts reparative gene activation post-myocardial infarction. Circ. Res. 131 (11), 893–908. 10.1161/circresaha.122.320488 36268709

[B99] Wang H. Yang L. Liu M. Luo J. (2023). Protein post-translational modifications in the regulation of cancer hallmarks. Cancer Gene Ther. 30 (4), 529–547. 10.1038/s41417-022-00464-3 35393571

[B100] Wang M. Huang Z. Li X. He P. Sun H. Peng Y. (2024). Apabetalone, a bet protein inhibitor, inhibits kidney damage in diabetes by preventing pyroptosis via modulating the p300/h3k27ac/plk1 axis. Pharmacol. Res 207, 107306. 10.1016/j.phrs.2024.107306 39002871

[B101] Wang Y. Li H. Jiang S. Fu D. Lu X. Lu M. (2024). The glycolytic enzyme pfkfb3 drives kidney fibrosis through promoting histone lactylation-mediated nf-κb family activation. Kidney Int 106 (2), 226–240. 10.1016/j.kint.2024.04.016 38789037

[B102] Wang K. Kong F. Han X. Zhi Y. Wang H. Ren C. (2025). Integrative multi-omics reveal NSUN2 facilitates glycolysis and histone lactylation-driven immune evasion in renal carcinoma. Genes Immun. 26 (4), 312–323. 10.1038/s41435-025-00336-4 40413354

[B103] Wang W. Chen Y. Cheng M. Wei L. Sang Y. Gao Y. (2026). Gentisic acid inhibits renal fibrosis by reprogramming glycolytic metabolism and regulating H3K18la. Bioorg Chem. 168, 109302. 10.1016/j.bioorg.2025.109302 41337826

[B104] Wei S. Gao Y. Dai X. Fu W. Cai S. Fang H. (2019). Sirt1-mediated hmgb1 deacetylation suppresses sepsis-associated acute kidney injury. Am. J. Physiol Renal Physiol 316 (1), F20–f31. 10.1152/ajprenal.00119.2018 30379096

[B105] Wei S. Dai Z. Wu L. Xiang Z. Yang X. Jiang L. (2025). Lactate-induced macrophage hmgb1 lactylation promotes neutrophil extracellular trap formation in sepsis-associated acute kidney injury. Cell Biol Toxicol 41 (1), 78. 10.1007/s10565-025-10026-6 40304798 PMC12043764

[B106] Wu X. Liu D. Gao X. Xie F. Tao D. Xiao X. (2017). Inhibition of brd4 suppresses cell proliferation and induces apoptosis in renal cell carcinoma. Cell Physiol. Biochem 41 (5), 1947–1956. 10.1159/000472407 28391274

[B107] Xiang T. Wang X. Huang S. Zhou K. Fei S. Zhou B. (2025). Inhibition of pkm2 by shikonin impedes tgf-β1 expression by repressing histone lactylation to alleviate renal fibrosis. Phytomedicine 136, 156324. 10.1016/j.phymed.2024.156324 39700636

[B108] Xie T. Yao L. Li X. (2024). Advance in iron metabolism, oxidative stress and cellular dysfunction in experimental and human kidney diseases. Antioxidants (basel) 13 (6), 659. 10.3390/antiox13060659 38929098 PMC11200795

[B109] Xiong J. He J. Zhu J. Pan J. Liao W. Ye H. (2022). Lactylation-driven mettl3-mediated rna m(6)a modification promotes immunosuppression of tumor-infiltrating myeloid cells. Mol. Cell 82 (9), 1660–1677.e1610. 10.1016/j.molcel.2022.02.033 35320754

[B110] Xu Z. Jia K. Wang H. Gao F. Zhao S. Li F. (2021). Mettl14-regulated pi3k/akt signaling pathway via pten affects hdac5-mediated epithelial-mesenchymal transition of renal tubular cells in diabetic kidney disease. Cell Death Dis 12 (1), 32. 10.1038/s41419-020-03312-0 33414476 PMC7791055

[B111] Xu L. Zhou Y. Wang G. Bo L. Jin B. Dai L. (2023). The udpase entpd5 regulates er stress-associated renal injury by mediating protein n-glycosylation. Cell Death Dis 14 (2), 166. 10.1038/s41419-023-05685-4 36849424 PMC9971188

[B112] Xu L. Zhang J. J. Xu W. T. (2026). Telitacicept alleviates IgA nephropathy by targeting PDHA1 lactylation to inhibit B cell metabolic reprogramming and lactate-mediated renal injury. Eur. J. Med. Res. 31, 527. 10.1186/s40001-026-04175-5 41776680

[B113] Yang J. Luo L. Zhao C. Li X. Wang Z. Zeng Z. (2022). A positive feedback loop between inactive vhl-triggered histone lactylation and pdgfrβ signaling drives clear cell renal cell carcinoma progression. Int. J. Biol Sci 18 (8), 3470–3483. 10.7150/ijbs.73398 35637958 PMC9134910

[B114] Yang F. Q. Liu M. Yang F. P. Che J. Li W. Zhai W. (2014). Vpa inhibits renal cancer cell migration by targeting hdac2 and down-regulating hif-1α. Mol. Biol Rep 41 (3), 1511–1518. 10.1007/s11033-013-2996-2 24390319

[B115] Yang K. Fan M. Wang X. Xu J. Wang Y. Tu F. (2022). Lactate promotes macrophage hmgb1 lactylation, acetylation, and exosomal release in polymicrobial sepsis. Cell Death Differ. 29 (1), 133–146. 10.1038/s41418-021-00841-9 34363018 PMC8738735

[B116] Yang Z. Yan C. Ma J. Peng P. Ren X. Cai S. (2023a). Lactylome analysis suggests lactylation-dependent mechanisms of metabolic adaptation in hepatocellular carcinoma. Nat. Metab 5 (1), 61–79. 10.1038/s42255-022-00710-w 36593272

[B117] Yang L. Wang X. Liu J. Liu X. Li S. Zheng F. (2023b). Prognostic and tumor microenvironmental feature of clear cell renal cell carcinoma revealed by m6a and lactylation modification-related genes. Front. Immunol 14, 1225023. 10.3389/fimmu.2023.1225023 37638005 PMC10450969

[B118] Ye Z. Sun Y. Yang S. Li L. Li B. Xia Y. (2025). Lgals3 promotes calcium oxalate crystal formation and kidney injury through histone lactylation-mediated fgfr4 activation. Adv. Sci (weinh) 12 (12), e2413937. 10.1002/advs.202413937 39903812 PMC11947994

[B119] Yu J. Chai P. Xie M. Ge S. Ruan J. Fan X. (2021). Histone lactylation drives oncogenesis by facilitating m(6)a reader protein ythdf2 expression in ocular melanoma. Genome Biol. 22 (1), 85. 10.1186/s13059-021-02308-z 33726814 PMC7962360

[B120] Yu F. Meng Y. Wang X. Chen J. He J. Yi X. (2025a). Protein lactylation of citrate synthase promotes the AKI-CKD transition by activating the NLRP3 inflammasome. Cell Rep. 44 (8), 116084. 10.1016/j.celrep.2025.116084 40748753

[B121] Yu H. Yin H. Mei Y. Bai X. Zhang C. Feng Z. (2025b). Nucleophosmin 1 lactylation in graft kidney induces ferroptotic trigger waves that exacerbate delayed graft function. Nat. Commun. 17 (1), 277. 10.1038/s41467-025-66991-3 41350284 PMC12783763

[B122] Yuan F. Huang J. Huang D. Li K. Zhou S. Zhou L. (2026). Identification of lactylation and its hub genes in contributing immune activation and renal allograft fibrosis by integrative bioinformatics and machine learning. Front. Immunol. 17, 1741864. 10.3389/fimmu.2026.1741864 41756296 PMC12932934

[B123] Zanfardino P. Amati A. Perrone M. Petruzzella V. (2025). The balance of MFN2 and OPA1 in mitochondrial dynamics, cellular homeostasis, and disease. Biomolecules 15 (3), 433. 10.3390/biom15030433 40149969 PMC11940761

[B124] Zeng J. Huang H. Zhang Y. Lv X. Cheng J. Zou S. J. (2023). Dapagliflozin alleviates renal fibrosis in a mouse model of adenine-induced renal injury by inhibiting tgf-β1/mapk mediated mitochondrial damage. Front. Pharmacol 14, 1095487. 10.3389/fphar.2023.1095487 36959860 PMC10028454

[B125] Zhang D. Tang Z. Huang H. Zhou G. Cui C. Weng Y. (2019). Metabolic regulation of gene expression by histone lactylation. Nature 574 (7779), 575–580. 10.1038/s41586-019-1678-1 31645732 PMC6818755

[B126] Zhang W. Guan Y. Bayliss G. Zhuang S. (2020). Class iia hdac inhibitor tmp195 alleviates lipopolysaccharide-induced acute kidney injury. Am. J. Physiol Renal Physiol 319 (6), F1015–f1026. 10.1152/ajprenal.00405.2020 33017186 PMC7792695

[B127] Zhang X. Chen J. Lin R. Huang Y. Wang Z. Xu S. (2024). Lactate drives epithelial-mesenchymal transition in diabetic kidney disease via the h3k14la/klf5 pathway. Redox Biol. 75, 103246. 10.1016/j.redox.2024.103246 38925041 PMC11255112

[B128] Zhang D. Gao J. Zhu Z. Mao Q. Xu Z. Singh P. K. (2025). Lysine L-lactylation is the dominant lactylation isomer induced by glycolysis. Nat. Chem. Biol. 21 (1), 91–99. 10.1038/s41589-024-01680-8 39030363 PMC11666458

[B129] Zheng T. Gu Y. P. Wang J. M. Huang T. T. Gou L. S. Liu Y. W. (2025). Lactate-triggered histone lactylation contributes to podocyte epithelial-mesenchymal transition in diabetic nephropathy in mice. Chem. Biol Interact 408, 111418. 10.1016/j.cbi.2025.111418 39922521

[B130] Zhou X. Chen H. Hu Y. Ma X. Li J. Shi Y. (2023). Enhancer of zeste homolog 2 promotes renal fibrosis after acute kidney injury by inducing epithelial-mesenchymal transition and activation of m2 macrophage polarization. Cell Death Dis 14 (4), 253. 10.1038/s41419-023-05782-4 37029114 PMC10081989

[B131] Zhou J. Zhang J. Xu F. Gao H. Wang L. Zhao Y. (2024). Ast-120 alleviates renal ischemia-reperfusion injury by inhibiting hk2-mediated glycolysis. Mol. Med 30 (1), 133. 10.1186/s10020-024-00902-y 39217289 PMC11365134

[B132] Zhu R. Ye X. Lu X. Xiao L. Yuan M. Zhao H. (2025). ACSS2 acts as a lactyl-CoA synthetase and couples KAT2A to function as a lactyltransferase for histone lactylation and tumor immune evasion. Cell Metab. 37 (2), 361–376.e367. 10.1016/j.cmet.2024.10.015 39561764

[B133] Zong Z. Xie F. Wang S. Wu X. Zhang Z. Yang B. (2024). Alanyl-tRNA synthetase, AARS1, is a lactate sensor and lactyltransferase that lactylates p53 and contributes to tumorigenesis. Cell 187 (10), 2375–2392.e2333. 10.1016/j.cell.2024.04.002 38653238

